# Parental alcohol and drug abuse and offspring mortality by age 10: a population-based register study

**DOI:** 10.1093/eurpub/ckac142

**Published:** 2022-09-28

**Authors:** Venla Berg, Ralf Kuja-Halkola, Lotfi Khemiri, Henrik Larsson, Paul Lichtenstein, Antti Latvala

**Affiliations:** Institute for Molecular Medicine Finland, University of Helsinki, Helsinki, Finland; Department of Medical Epidemiology and Biostatistics, Karolinska Institutet, Solna, Sweden; Institute of Criminology and Legal Policy, University of Helsinki, Helsinki, Finland; Population Research Institute, Väestöliitto, Helsinki, Finland; Department of Medical Epidemiology and Biostatistics, Karolinska Institutet, Solna, Sweden; Department of Medical Epidemiology and Biostatistics, Karolinska Institutet, Solna, Sweden; Department of Medical Epidemiology and Biostatistics, Karolinska Institutet, Solna, Sweden; School of Medical Sciences, Örebro University, Örebro, Sweden; Department of Medical Epidemiology and Biostatistics, Karolinska Institutet, Solna, Sweden; Institute for Molecular Medicine Finland, University of Helsinki, Helsinki, Finland; Department of Medical Epidemiology and Biostatistics, Karolinska Institutet, Solna, Sweden; Institute of Criminology and Legal Policy, University of Helsinki, Helsinki, Finland

## Abstract

**Background:**

Parental substance abuse (SA) of alcohol and drugs is associated with offspring mortality, including sudden infant death syndrome (SIDS), in infancy, but research on cause-specific mortality and mortality in later childhood is scarce.

**Methods:**

Using population-based register data on all births in Sweden in 1973–2013 (*N* = 4.2 million) and Cox regressions, we examined the associations of mother’s and father’s SA registered between 2 years before and 12 years after the child birth with offspring all-cause and cause-specific mortality in infancy and childhood.

**Results:**

Parental SA was associated with increased offspring all-cause and natural-cause mortality in infancy, but not in the neonatal period, and with external-cause mortality in ages 1–9. Risk of SIDS was 130–280% higher in infants with parental SA compared to infants with no parental SA. Adjusting for parental socioeconomic and immigrant status and severe psychiatric disorders, paternal SA was associated with 66% higher mortality due to communicable diseases and infections in infancy, and both maternal and paternal SA were associated with 40–174% higher mortality due to accidents in infancy and in ages 1–9. The associations between parental SA and offspring mortality were similar for male and female offspring.

**Conclusions:**

Child mortality is rare in contemporary Sweden, and parental SA has variable associations with elevated offspring mortality throughout the first 10 years of life, excluding the neonatal period, which is indicative of insufficient recognition of children at risk. Preventive measures should be long-term and targeted to both parental and offspring behaviour.

## Introduction

An estimated every 10th child in Western countries has a parent who misuses alcohol or drugs, and these children are known to be at risk of emotional, physical and socioeconomic adversities.^1^ Most alarmingly, offspring of substance-abusing parents have an elevated mortality risk, but the details of this association are still unclear. Firstly, while parental substance abuse (SA) is often a lasting condition likely to affect offspring throughout childhood,^1^ knowledge of its association with offspring mortality beyond infancy is very limited. Most studies have examined mother’s alcohol and/or illicit drug use during or before pregnancy and offspring all-cause mortality in the neonatal period or infancy,^2–12^ whereas research beyond infancy is rare and has often focused on specific aspects of parental SA, such as foetal alcohol syndrome or methadone treatment participants.^13–16^ Secondly, research on cause-specific mortality is sparse, even though information on causes of death would be crucial for prevention. A number of studies have found the risk of sudden infant death syndrome (SIDS) to be elevated in offspring of mothers with prior substance use problems or substance use during pregnancy.^3^^,^^10^^,^^17–19^ Only two studies have examined cause-specific mortality in later childhood, one finding elevated natural and unnatural-cause mortality by age 6 among offspring of opioid-dependent parents^15^ and the other finding no association with cancer mortality by age 16 among offspring of heavy drinkers.^20^

Thirdly, only few prior studies have investigated the role of parental SA beyond confounding factors, such as low socioeconomic status (SES) and smoking.^7^^,^^10^^,^^14^^,^^18^ Since substance use problems can be successfully targeted by interventions [e.g. Ref. (21)] it would be important to examine their specific contribution independently of other factors [e.g. Ref. (22)] Finally, far less attention has been paid to the role of paternal SA as compared to maternal, in line with other parental factors in child health [e.g. Ref. (23)] and with paediatric health care systems’ typical focus on the mothers [e.g. Ref. (24)]. In these studies, paternal SA has been associated with elevated offspring mortality in infancy, albeit less strongly than maternal SA.^15^^,^^16^^,^^18^^,^^19^

In the current study, we examined the associations between parental SA and offspring mortality in childhood using whole-population register data from Sweden. We studied offspring all-cause and cause-specific mortality addressing the role of both maternal and paternal SA and taking into account some other risk factors.

## Methods

### Data

We linked several Swedish nationwide registers using a unique personal identity number assigned at birth or immigration. The Multi-Generation Register, as part of the Total Population Register, identifies biological and adoptive parents of each individual born since 1932 and living in Sweden at any time since 1961 and was used to identify family pedigrees. Register data for parental SA start on 1 January 1973 and were available until the end of 2013. The cohort included all individuals born in Sweden in 1973–2013, for whom both parents could be identified (*N* = 4 198 952).

Information on parental substance use disorders and other mental disorders was obtained from the National Patient Register, which contains detailed hospitalization episodes since 1973 and outpatient treatments in specialist care since 2001. Information on parental alcohol and drug-related criminal convictions were obtained from the National Crime Register. Parents’ and offspring’s dates and countries of birth were obtained from the Total Population Register and from the Medical Birth Register. Data on parental education and income were available from National Censuses undertaken in 1970, 1975, 1980 and 1985, and from the Longitudinal Integration Database for Health Insurance and Labour Market Studies from 1990 onwards. Causes and dates of death were obtained from the Cause of Death Register, and emigration dates from the Migration Register. Gestational age and maternal smoking during pregnancy were available in the Medical Birth Register. Stillbirths were not included in the analyses. Register linkages for the current study were approved by the Regional Ethical Review Board of Stockholm. No informed consent was required for the anonymized register data. The data underlying this article were provided by different Swedish official registers under license for the current study. Data are available for all researchers after ethical vetting and application to the appropriate registers.

### Measures

Parental SA was defined as diagnoses of alcohol and drug-related psychiatric disorders and diagnoses of somatic illnesses caused by excessive alcohol use (complete list of codes in Supplementary table S1). In addition, we included convictions of alcohol or drug-related crimes (Supplementary table S1). Registered dates are not precise indicators of the timing of actual SA problems, and any event during the lifetime may indicate long-term problems. However, to increase the likelihood of the focal child being affected by parental SA in their childhood, we examined all parental SA registrations within a period between 2 years before the focal child birth and 12 years after the birth. Of mothers and fathers who had at least one SA registration during the lifetime, 39% and 45%, respectively, also had an SA registration during this period. In the main analyses, we used a binary variable with having no diagnoses or convictions during this time period coded as ‘0’ and having at least one diagnosis or conviction coded as ‘1’. Additionally, we utilized the date of the first diagnosis or criminal conviction in sensitivity analyses (see Statistical analyses section). Further, to maximize statistical power when examining rare causes of death, we also used parental SA registrations during the lifetime (similarly coded as a binary variable) as a predictor in some sensitivity analyses.

Neonatal deaths were defined as deaths within the first 27 days after birth, infant deaths as deaths that happened between 28 days and 12 months, and childhood deaths as deaths occurring between the first and 10th birthday. Causes of death were defined based on underlying cause of death, and categorized into natural-cause mortality and external-cause mortality. In addition, we examined selected specific causes of death: communicable diseases and acute infections, benign and malign neoplasms, congenital malformations, accidents and homicides. Additionally, we examined SIDS mortality as a separate category by age 1 without a division into neonatal and infant mortality, to allow comparison with previous research and because SIDS mortality is rare in the neonatal period. The complete list of diagnoses included in each category is shown in Supplementary table S2.

Parental SES was defined as parents’ highest education (<9, 9–12 and 12+ years) and father’s income (average income decile in child age 0–10; see details in Supplementary text S1). Parental immigration status was coded as ‘0’ if the parent was born in Sweden and as ‘1’ if not. Parental severe psychiatric disorders were defined as lifetime diagnoses of schizophrenia and bipolar disorder (list of ICD codes in Supplementary table S2), and coded as ‘1’ if the parent had a lifetime registration of any of these diagnoses, and otherwise as ‘0’. Maternal smoking during pregnancy was coded as ‘1’ if the mother reported smoking at either prenatal care registration or at gestation week 30–32 and as ‘0’ if the mother reported not smoking at both of these times. Preterm birth was divided into two categories, early preterm birth (<34 weeks of gestation) and late preterm birth (≥34 and <37 weeks).

### Statistical analyses

Neonatal mortality was examined using logistic regression. The timing of death from age 28 days onwards was examined with Cox proportional hazards models, with attained age as the underlying time-scale. Those not deceased contributed person-time at risk until their 10th birthday, the end of follow-up (end of 2013) or emigration, whichever occurred first. All models were adjusted for child’s sex and birth year, mother’s age at offspring’s birth and quadratic mother’s age at offspring’s birth. To assess the independent role of parental SA in offspring mortality, we further examined the associations adjusting for parental education, income, immigrant status and severe mental disorders. Neonatal mortality, infant mortality and mortality in childhood (deaths in ages 1–9; see the proportional hazard tests for this period in Supplementary text S2 and figures S1–S3) were modelled separately. Additionally, SIDS mortality by age 1 was examined by logistic regression in separate analyses, and in these analyses, we controlled for preterm birth and maternal smoking during pregnancy (missing data in them were included in the analyses as a separate category).

The similarity in the associations between parental SA and offspring mortality in male and female offspring was examined by models which included the main effects of parental SA and offspring sex, and an interaction term between them. Since we defined parental SA as registrations between 2 years before and 12 years after the focal child’s birth, the first observed parental SA registration may have occurred after the child’s death. We examined the timing of parental SA registrations relative to child birth and death, as well as the number of parental SA registrations by offspring’s timing of death in Supplementary analyses. The possibility of reverse causation (death of a child causing SA in parents) was also assessed by Cox regressions with time-varying parental SA as the exposure. In these analyses, parental SA was recorded as ‘0’ before the first date of diagnosis or criminal conviction, and ‘1’ after that. Finally, because restricting the time frame of registered parental SA may also exclude parents with prominent SA problems in the child’s childhood, we conducted sensitivity analyses using lifetime parental SA as exposure (i.e. all parents who had at least one SA registration during the follow-up were coded as having SA). All analyses were performed with Stata 14. The analysis plan was not pre-registered.

## Results

Of offspring, 0.8% had a mother, 4.7% a father and 0.5% both a mother and a father with registered SA in their childhood (table 1); 2.1% had a mother, 10.2% a father and 1.2% both a mother and a father with at least one SA registration at any point during the study period. Parental SA was related to several other childhood risk factors, including parental severe psychiatric disorders, lower SES and maternal smoking during pregnancy (table 1). By the 10th birthday, 0.90% of offspring who had at least one parent with SA had died, compared to 0.52% of offspring with no parental SA.

**Table 1:** Sample characteristics by parental SA

	No parental SA	Maternal SA	Paternal SA	Both parents’ SA
*N* (%)	3 948 809 (94.0)	33 887 (0.8)	196 351 (4.7)	19 905 (0.5)
Neonatal deaths (*n*, %)	10 531 (0.27)	103 (0.30)	518 (0.26)	46 (0.23)
Natural-cause	10 324 (0.26)	102 (0.30)	505 (0.26)	46 (0.23)
External-cause	24 (0.00)	0 (0.00)	2 (0.00)	0 (0.00)
Infant deaths (*n*, %)	5620 (0.14)	108 (0.32)	456 (0.23)	77 (0.39)
Natural-cause	5324 (0.13)	97 (0.29)	422 (0.21)	69 (0.35)
External-cause	182 (0.00)	9 (0.03)	22 (0.01)	5 (0.03)
Deaths in age 1–9 (*n*, %)	4988 (0.13)	67 (0.20)	371 (0.19)	101 (0.20)
Natural-cause	3561 (0.08)	34 (0.10)	215 (0.11)	21 (0.11)
External-cause	1279 (0.03)	32 (0.09)	146 (0.07)	19 (0.10)
SIDS (*n*, %)	1600 (0.04)	53 (0.16)	195 (0.10)	43 (0.22)
Preterm birth (%)				
Late (3–37 weeks)	4.1	6.4	5.0	7.0
Early (<34 weeks)	1.4	2.4	1.8	2.9
Missing	2.2	2.1	2.2	2.5
SDP (%)				
No	61.2	38.0	39.6	17.7
Yes	10.9	38.0	27.3	53.2
Missing (%)	27.9	24.0	33.1	29.1
Mother’s age at birth (mean, SD)	29.3 (5.2)	27.5 (5.8)	26.9 (5.7)	26.4 (5.8)
Mother’s birth year (mean, SD)	1964.1 (11.7)	1966.0 (11.6)	1963.7 (11.5)	1965.9 (12.1)
Father’s birth year	1961.3 (12.0)	1962.6 (12.3)	1960.4 (12.0)	1962.2 (12.6)
Severe mental disorder (%)				
Mother’s	1.0	10.7	2.2	8.0
Father’s	0.7	1.3	3.4	3.6
Mother immigrant (%)	15.9	15.6	20.9	13.5
Father immigrant (*n*, %)	16.3	17.0	25.4	17.7
Mother’s education (%)				
Low	11.5	28.2	22.6	40.7
Middle	45.1	53.3	53.9	49.0
High	42.4	17.8	22.0	8.8
Missing	0.9	0.8	1.5	1.6
Father’s education				
Low	17.2	24.2	32.7	40.0
Middle	47.8	56.1	53.8	52.3
High	34.0	18.2	11.4	5.0
Missing	1.0	1.5	2.0	2.6
Father’s income decile (mean, SD)	6.4 (2.2)	5.7 (2.3)	4.5 (2.2)	3.5 (1.9)

SD, standard deviation; SA, substance abuse; SIDS, sudden infant death syndrome; SDP, maternal smoking during pregnancy.

Supplementary figure S4 shows offspring mortality by parental SA throughout the observation period. The absolute numbers of deaths by offspring age and cause of death, and the proportions of different causes of death within age groups are shown in Supplementary figures S5 and S6, respectively. The vast majority of all offspring deaths occurred within the first year of offspring’s life, and of these, two-thirds were neonatal deaths. In infancy and early childhood, the majority of deaths were due to natural causes, and external causes of death increased with age.

Parental SA was not associated with neonatal mortality, apart from an association between father’s SA and lower risk of mortality due to congenital malformations (table 2), which persisted when controlling for mother’s SA and parental socioeconomic and immigrant status and severe psychiatric disorders [odds ratio (OR) = 0.83, 95% confidence interval (CI) 0.71–0.98, *P* = 0.029].

**Table 2:** ORs and 95% CIs from logistic regression analyses predicting neonatal offspring all-cause and cause-specific mortality by parental SA

	OR (95% CI)	*P*-value
All-cause mortality		
Mother’s SA	1.09 (0.92, 1.28)	0.311
Father’s SA	0.94 (0.86, 1.02)	0.153
Natural-cause mortality		
Mother’s SA	1.10 (0.94, 1.30)	0.249
Father’s SA	0.92 (0.85, 1.01)	0.072
Infectious diseases		
Mother’s SA	1.45 (0.64, 3.25)	0.370
Father’s SA	0.81 (0.49, 1.34)	0.405
Neoplasms		
Mother’s SA	a	
Father’s SA	1.39 (0.50, 3.88)	0.530
Congenital malformations		
Mother’s SA	0.74 (0.53, 1.03)	0.072
Father’s SA	0.82 (0.70, 0.95)	0.009
External-cause mortality		
Mother’s SA	a	
Father’s SA	1.49 (0.35, 6.43)	0.591

*Note*: All models control for offspring’s sex and birth year, mother’s age at offspring’s birth and quadratic mother’s age at offspring’s birth.

a Too few cases for model estimation. External mortality in the neonatal period was rare (26 cases, none in the maternal SA group), preventing the estimation of mortality due to accidents and homicides. Because the associations between parental SA and neonatal mortality were weak and not statistically significant, no models with further adjustments were run, except for the association between father’s SA and congenital malformations (see text for the results).

CI, confidence interval; OR, odds ratio; SA, substance abuse.

In infancy, mother’s and father’s SA were associated with a 49–381% increased risk of all-cause, natural-cause and external-cause mortality, and of infectious disease and accident mortality specifically, but not mortality related to neoplasms, congenital malformations or violent assaults (table 3, Model 1). In ages 1–9, parental SA was associated with a 31–453% increased risk of all-cause, infectious disease and external-cause mortality including accident and homicide mortality. Infectious disease mortality was only related to father’s SA and homicide mortality only to mother’s SA (table 4, Model 1).

**Table 3:** All-cause and cause-specific mortality in infancy by parental SA

	Model 1		Model 2	
	HR (95% CI)	*P*-value	HR (95% CI)	*P*-value
**All-cause mortality**				
Mother’s SA	2.23 (1.93, 2.59)	<0.001	1.75 (1.48, 2.07)	<0.001
Father’s SA	1.53 (1.40, 1.67)	<0.001	1.26 (1.14, 1.39)	<0.001
**Natural-cause mortality**				
Mother’s SA	2.13 (1.82, 2.49)	<0.001	1.71 (1.44, 2.04)	<0.001
Father’s SA	1.49 (1.36, 1.64)	<0.001	1.24 (1.12, 1.38)	<0.001
Infectious diseases				
Mother’s SA	2.32 (1.48, 3.63)	<0.001	1.52 (0.90, 2.59)	0.121
Father’s SA	1.97 (1.53, 2.53)	<0.001	1.66 (1.25, 2.21)	<0.001
Neoplasms				
Mother’s SA	a		a	
Father’s SA	0.84 (0.37, 1.91)	0.672	0.87 (0.35, 2.18)	0.769
Congenital malformations				
Mother’s SA	1.05 (0.72, 1.52)	0.813	1.02 (0.68, 1.53)	0.922
Father’s SA	0.93 (0.77, 1.13)	0.479	0.88 (0.71, 1.08)	0.214
**External-cause mortality**				
Mother’s SA	4.81 (2.78, 8.31)	<0.001	2.85 (1.48, 5.49)	0.002
Father’s SA	2.15 (1.42, 3.24)	<0.001	1.46 (0.91, 2.34)	0.117
Accident				
Mother’s SA	4.24 (2.16, 8.35)	<0.001	2.74 (1.28, 5.87)	0.009
Father’s SA	2.53 (1.62, 3.97)	<0.001	1.87 (1.12, 3.13)	0.017
Homicide				
Mother’s SA	2.16 (0.29, 16.16)	0.452	1.18 (0.14, 9.76)	0.878
Father’s SA	1.06 (0.25, 4.57)	0.938	0.76 (0.16, 3.57)	0.727

Model 1 controls for offspring’s sex and birth year, mother’s age at offspring’s birth and quadratic mother’s age at offspring’s birth.

Model 2 controls for Model 1 and father’s income, co-parent’s SA, and mother and father’s education, immigrant status, and severe mental disorders.

a Not enough cases for model estimation.

CI, confidence interval; HR, hazard ratio; SA, substance abuse.

**Table 4:** All-cause and cause-specific mortality in ages 1–9 by parental SA

	Model 1		Model 2	
	HR (95% CI)	*P*-value	HR (95% CI)	*P*-value
**All-cause mortality**				
Mother’s SA	1.45 (1.20, 1.75)	<0.001	1.17 (0.94, 1.46)	0.155
Father’s SA	1.31 (1.18, 1.45)	<0.001	1.09 (0.97, 1.22)	0.157
**Natural-cause mortality**				
Mother’s SA	1.08 (0.83, 1.41)	0.571	1.01 (0.75, 1.35)	0.973
Father’s SA	1.08 (0.95, 1.24)	0.240	0.96 (0.83, 1.11)	0.576
Infectious diseases				
Mother’s SA	1.34 (0.71, 2.50)	0.365	1.20 (0.61, 2.35)	0.604
Father’s SA	1.50 (1.11, 2.03)	0.009	1.30 (0.92, 1.83)	0.130
Neoplasms				
Mother’s SA	1.09 (0.68, 1.77)	0.718	1.04 (0.61, 1.79)	0.876
Father’s SA	0.94 (0.73, 1.21)	0.632	0.87 (0.65, 1.15)	0.313
Congenital malformations				
Mother’s SA	0.99 (0.56, 1.76)	0.983	0.99 (0.54, 1.81)	0.964
Father’s SA	1.03 (0.79, 1.36)	0.809	0.91 (0.67, 1.23)	0.534
**External-cause mortality**				
Mother’s SA	2.51 (1.90, 3.33)	<0.001	1.53 (1.10, 2.15)	0.013
Father’s SA	1.90 (1.61, 2.24)	<0.001	1.37 (1.14, 1.66)	0.001
Accidents				
Mother’s SA	2.32 (1.70, 3.16)	<0.001	1.48 (1.03, 2.13)	0.034
Father’s SA	1.87 (1.57, 2.23)	<0.001	1.40 (1.14, 1.71)	0.001
Homicides				
Mother’s SA	5.53 (2.68, 11.44)	<0.001	2.53 (0.96, 6.70)	0.061
Father’s SA	1.72 (0.92, 3.25)	0.091	0.98 (0.46, 2.07)	0.951

Model 1 controls for offspring’s sex and birth year, mother’s age at offspring’s birth and quadratic mother’s age at offspring’s birth.

Model 2 controls for Model 1 and father’s income, co-parent’s SA, and mother and father’s education, immigrant status, and severe mental disorders.

CI, confidence interval; HR, hazard ratio; SA, substance abuse.

The associations between parental SA and offspring all-cause, natural-cause and external-cause mortality in infancy were attenuated, but still prominent in most cases (24–185% increased risks) when mutually adjusting for mother’s and father’s SA and parental SES, immigration and severe psychiatric disorders (table 3, Model 2; for the full results, see Supplementary tables S3–S5). The associations between maternal SA and infectious disease mortality and paternal SA and external-cause mortality were attenuated to a statistically non-significant level. In childhood, ages 1–9, parental SA remained a significant predictor for offspring overall external-cause and accident mortality (37–53% increased risks) when adjusting for other childhood risk factors, but the associations between parental SA and all-cause mortality, paternal SA and infectious disease mortality, and maternal SA and homicide mortality were statistically non-significant (table 4, Model 2; for the full results, see Supplementary tables S6–S8). For these outcomes, a substantial part of the increased offspring mortality seemed to relate to confounding risk factors, even though some of the statistically non-significant findings may result from lack of statistical power rather than lack of an association per se (see Sensitivity analyses with a more comprehensive parental SA definition).

SIDS mortality by age 1 was associated with mother’s (OR = 3.77, 95% CI 3.07–4.64) and father’s SA (OR = 2.26, 95% CI 1.97–2.60; Supplementary table S9). The associations with SIDS remained prominent, although attenuated, when mutually adjusting for both parents’ SA, mother’s smoking during pregnancy and preterm birth (OR = 2.11, 95% CI 1.70–2.62; and OR = 1.59, 95% CI 1.37–1.83 for mother’s and father’s SA, respectively; Supplementary table S9). Mortality was higher in male than female offspring in all ages and for most causes of death, but the associations between parental SA and offspring mortality were similar for male and female offspring (Supplementary table S10).

### Sensitivity analyses

We examined the timing of parental SA events in more detail to address the issues of reverse causality and low statistical power in relation to very rare causes of death. Of mothers and fathers with at least one registered SA event between 2 years before and 12 years after the focal child birth, only 14% and 12%, respectively, only had events before the child’s birth. This indicates that parental SA problems are often long-term, possibly affecting the child throughout their childhood. Further, only a minority of parents with registered SA during the lifetime had them exclusively very long before or very long after the child’s birth—most had SA registrations during the child’s childhood (Supplementary figure S7). Among parents whose child deceased within ten years from birth, registered SA events were concentrated around the time of the child’s death, indicating prominent problems at that period of the child’s life (Supplementary figure S8). There was no marked increase in parental SA registrations after the child’s death, suggesting that reverse causality had little effect on the associations between parental SA and child mortality. This finding was confirmed with analyses using time-varying parental SA as a predictor, which was similarly associated with offspring mortality (Supplementary table S11; only the main causes of death were examined) as was parental SA between 2 years before and 2 years after the child’s birth.

Since timing of parental SA registrations had little effect on the associations, to capture all potential parental SA problems and maximize statistical power to examine especially rare causes of death, we repeated the main analyses using a lifetime parental SA definition. The results were similar to the main results (Supplementary tables S12 and S13). Some of the non-significant results in the main analyses were similar in magnitude but statistically significant in these sensitivity analyses; specifically, in infancy, (i) the association between maternal SA and offspring infectious disease mortality, (ii) the association between paternal SA and external-cause mortality in the models controlling for additional risk factors; and, in ages 1–9, associations between maternal SA, (iii) offspring infectious disease mortality in models not controlling and models controlling for additional risk factors and (iv) homicide mortality in the models controlling for additional risk factors (Supplementary tables S12 and S13).

## Discussion

In this Swedish population study, we found the absolute mortality risk in childhood to be very low (by the 10th birthday, 0.55% of offspring had died). We also found parental SA to be associated with relatively higher, in some cases substantially increased, risks of offspring mortality in infancy and childhood, excluding the neonatal period. Mother’s SA was associated with overall all-cause, natural-cause and external-cause mortality, and specifically accident mortality in infancy, and with overall external-cause and accident mortality in ages 1–9. Father’s SA was associated with overall all-cause and natural-cause mortality, and specifically infectious disease and accident mortality in infancy, and with overall external-cause and accident mortality in ages 1–9. Importantly, these associations were attenuated, but not eliminated, when controlling for parental SES and severe psychiatric diseases, suggesting that parental SA is an independent risk factor for child mortality above these known risk factors.^16^^,^^22^ For both natural-cause and external-cause mortality, we found a higher relative mortality risk for maternal as compared to paternal SA. Our results differ somewhat from a previous study using Danish register data, where parental alcohol and drug use disorders were not systematically associated with offspring mortality by age 15.^16^ However, their study was limited to first inpatient admission diagnoses in parents, leaving later substance use disorders and other possible indicators of SA undetected, and only examined all-cause mortality.

We also replicated previous findings concerning elevated SIDS mortality among offspring of substance-abusing parents.^3^^,^^10^^,^^17–19^ In addition, we found that the elevated risk of SIDS persevered when controlling for perinatal risk factors, further adding to the growing body of evidence that parental SA constitutes an independent risk for SIDS. Also, the risk seems not to be only related to maternal substance use during pregnancy but also with later and/or earlier substance use in mothers, as well as with father’s SA problems.

The higher infant mortality due to infections and communicable diseases in offspring of parents with SA is a novel and somewhat surprising finding in a contemporary welfare state with highly developed health care. The mechanisms behind the association between parental SA and offspring infectious disease mortality are not known, but could possibly involve negative effects of parental SA on the immune system, or parenting behaviour. For instance, prenatal alcohol exposure may cause long-lasting altered immune system functioning,^25^ resulting in children being more vulnerable to certain infections. In addition, early recognition and treatment of sepsis in children increases survival rates.^26^ Continued parental SA in offspring’s infancy and childhood,^1^ or parental psychiatric co-morbidity with SA, such as attention-deficit,^27^ may compromise the parents’ ability to monitor and attend to their children’s medical needs. Future studies on clinical patient cohorts are needed to understand the underlying mechanisms of the observed association.

Offspring of parents with SA problems were also at higher risk of external mortality due to accidents in infancy and childhood. The association with accident mortality was stronger in infancy than in later childhood, suggesting that the underlying mechanisms may vary with offspring age. In infancy and early childhood, accident mortality may be more strongly related to parental behaviour, whereas heritable tendencies towards risky behaviour in offspring^28^ presumably become more important as the children gain more independence. Notably, offspring of mothers with SA also had 450% higher homicide mortality in ages 1–9 in the model only adjusted for demographic covariates, whereas the association was attenuated and imprecisely estimated in the fully adjusted model. As the large majority of child homicides is intrafamilial,^29^ this result suggests that high-risk families are not always recognized, and more efforts are needed to prevent such tragic events.

With whole-population register data and a broad definition of parental SA, we were able to examine in detail the extremely rare outcome of childhood mortality in a contemporary welfare setting. However, register information on SA was restricted to medical diagnoses and criminal convictions, leaving especially milder cases undetected. Our results are best interpreted as pertaining to severe parental SA, and represent a conservative estimate of the associations between parental SA and offspring mortality, since the group treated as non-exposed in these analyses is likely to include many parents with SA problems as well. Further, the temporal order of parental SA and offspring mortality cannot be reliably assessed with register data because the date of diagnosis or conviction is not informative of how long the problem has persisted. In some cases, offspring death may cause substance-abusing behaviour in parents who have not previously manifested such problems. However, sensitivity analyses examining the timing of parental registered SA events, and with time-varying parental SA indicated a low likelihood for reverse causation affecting the observed associations to a substantial degree. Another caveat in our study is that as a parent not only provides for the childhood environment but also transfers 50% of their genes to their offspring, the possibility of genetic confounding in any associations between parents’ characteristics and children’s outcomes is apparent,^30^ in addition to other forms of residual confounding likely affecting our results. Future studies should further look into the genetic and environmental mechanisms underlying the associations between parental SA and child mortality.

Based on our results, offspring of parents with SA problems have an increased risk of all-cause and natural-cause mortality in infancy, and accident mortality throughout childhood, even when taking into account other risk factors. The risk extends to infectious disease and external mortality, beyond the previously reported increased risk of SIDS. Health care, educational and social service staff should be aware of this rare, but extremely serious outcome when working with parents with SA problems, throughout the children’s childhood. Preventive measures need to be directed to whole families, targeting both parental and offspring behaviour.

## Supplementary data

Supplementary data are available at *EURPUB* online.

## Funding

This research was funded by the research funds of the University of Helsinki, Academy of Finland (308698), the Swedish Research Council for Health, Working Life and Welfare, Stockholm County Council (20170264) and the Swedish Research Council. The funding sources had no role in the design and conduct of the study; collection, management, analysis and interpretation of the data; preparation, review or approval of the manuscript; and decision to submit the manuscript for publication.

*Conflicts of interest*: H.L. reported receiving grants and personal fees from Shire/Takeda and personal fees from Evolan, all outside the submitted work. The other authors declare no competing interests.

Key points

- In this Swedish population study, we found both maternal and paternal substance abuse (SA) to be associated with increased risks of offspring all-cause and natural-cause mortality in infancy, and with external-cause mortality through ages 1–9.
- Paternal SA was associated with increased infectious disease mortality in infancy, and both maternal and paternal SA with accident mortality in infancy and in ages 1–9.
- The associations were attenuated, but not eliminated, when controlling for parental socioeconomic status and severe psychiatric diseases, suggesting that parental SA is an independent risk factor for child mortality above these known risk factors.

## Supplementary Material

ckac142_Supplementary_Data
