# Supplementary material for: Parental alcohol and drug abuse and offspring mortality by age 10: a population-based register study
Source: Eur J Public Health. 2022 Sep 28;32(6):933–8. doi: 10.1093/eurpub/ckac142 (PMC9713384; doi:10.1093/eurpub/ckac142)
Supplement: ckac142_Supplementary_Data [file ckac142_supplementary_data.docx]

Supplementary material for

Parental alcohol and drug abuse and offspring mortality by age 10: A population-based register study

Berg, Venla; Kuja-Halkola, Ralf; Khemiri, Lotfi; Larsson, Henrik; Lichtenstein, Paul; Latvala, Antti

| Table S1. ICD codes (according to Swedish ICD versions) and criminal convictions used to define substance abuse. | | | |
| --- | --- | --- | --- |
| Diagnosis | ICD 8 | ICD 9 | ICD 10 |
| Alcohol use disorders | 291*; 303*; 261,00 | 291*; 292*; 303*; 305A | F10* |
| Drug use disorders | 304* | 304*; 305X | F11*-F16*; F18*-F19* |
| Neurological conditions caused by alcohol | - | 357F | G31.2 |
| Alcoholic cardiomyopathy | - | 425F | I42.6 |
| Alcohol gastritis | - | 535D | K29.2 |
| Alcohol-related chronic pancreatitis | - | - | K86.0 |
| Alcohol-related pseudo-Cushing's syndrome | - | - | E24.4 |
| Alcohol-related myopathy | - | - | G72.1 |
| Alcohol-related niacin deficiency | 262,00 | - | - |
| Alcohol-related liver diseases | 571,00 & 571,01 | 571A-D | K70* |
| Drug-related liver diseases | 070,03 | - | - |
| Toxic effect of alcohol | 980* | 980* | T51* |
| Toxic effect of drugs | - | - | T40* |
| Contact with social workers by a person with alcohol dependency history | - | V79B | - |
|  |  |  |  |
| Criminal conviction | Definition | | |
| Possession of illegal substances for personal use, supply, manufacture, or consumption | Narcotic Drugs Act (1968:64) | | |
| Driving under the influence of alcohol and/or illicit substances | Act 1951:649 | | |

*Any code starting with the said.

| Table S2. ICD codes used to classify causes of death and parental severe psychiatric morbidity. | | | |
| --- | --- | --- | --- |
|  | ICD 8 | ICD 9 | ICD 10 |
| **Causes of death** |  |  |  |
| **Natural-cause mortality (in addition to all sub-category codes)** | 135*; 240-290*; 292-302*; 304-315*; 321-322*; 324-458*; 490-528,0; 528,2-570*; 571,9-738*; 760-794* | 040B; 099C; 135-136A; 240-273B; 273D-290*; 292-302*; 304*; 306-319*; 330-357E; 357G-425E; 425H-459*; 467-478*; 490-535C; 535E-570*; 571E-739; 760A-H; 760X-779E; 779G-790C; 790E-797*; 798B-X; 799A | D50-D89*; E*; F00-F09*; F11-F99*; G10-G31.1; G31.8-G62.0; G62.2-G72.0; G72.2-I42.5; I42.7-I99*; J23-J64*; J66-J84.8; J85-K29.1; K29.3-K67*; K71-K85.1; K85.3-K85.9; K86.1-O35.3; O35.5-P04.2; P04.4-P96*; R00-R53-R94* |
| Sudden infant death syndrome | 795,00; 796,20 | 798A | R95 |
| Infections and communicable diseases | 000-134*; 136*; 275,9*; 320*; 323*; 46-48*; 528,1* | 001-040A; 040C-099B; 099D-134*; 136B-139*; 320-326*; 460-466*; 480-487*; 771C; 790H | A*; B*; J00-J06*; J09-J18*; J20-J22*; J65*; J84.9; G00-G09* |
| Neoplasms, malign and benign | 140-239*; 275,50 | 140-237G; 237X-239*; 273C | C00-D48* |
| Congenital malformations | 74-759* | 237H; 740-759* | Q00-Q85*; Q86.1-Q99* |
| **External-cause mortality (in addition to all sub-category codes)** | 291*; 303*; 571,00; 571,01; E860; E930-E936; E947; E949-E959; E970-E999 | 291*; 303*; 305A; 305X; 357F; 425F; 535D; 571A-D; 760W; 779F; E860*; E87*; E93-E95*; E97-E99* | F10*; G31.2; G62.1; G72.1; I42.6; K29.2; K70*; K85.2; K86.0; O35.4; P04.3; Q86.0; X45*; X60-X84*; Y10-Y84*; Y87.0; Y87.2; Y88-Y89* |
| Accidents | E800-E859; E861-E946; E948 | E800-E858; E861-E869; E880-E929 | V01-X44*; X46-X59*; Y85-Y86* |
| Homicides | E96* | E96* | X85-Y09*; Y87.1 |
| **Parental psychiatric comorbidity** |  |  |  |
| Schizophrenia | 295* | 295* | F20* |
| Bipolar disorder | 296,1*; 296,2*; 296,3*; 296,8* | 296C, D, DC & E | F31* |
| Note. Classification of causes of death is based on the classification of Statistics Finland (available at http://www.stat.fi/til/ksyyt/ksyyt_2018-11-12_luo_001.pdf; downloaded on 11 August 2020), with some categories combined and modified to Swedish ICD codes. *Any code starting with the said. | | | |

**Supplementary Text S1. Father’s income measure**

Parental income was defined as father’s yearly net income during the first ten years of the child’s life, categorized into deciles within each year, and then averaged across the ten years, resulting in a continuous measure ranging from 1–10. If information on income was missing for more than 5 years, this variable was set to missing (16% of fathers had missing income data).

**Supplementary Text S2. Testing the proportional hazards assumption.**

Because neonatal and infant mortality were much more prevalent than later mortality, they were analysed as separate time periods. Neonatal mortality was assessed with logistic regression and infant mortality and mortality in ages 1–9 with Cox regression. The Kaplan-Meier curves for all age periods are shown in Figs. S1–S3. For all-cause mortality in infancy and ages 1–9, we examined the proportional hazards assumption of Cox regression by (i) investigating interaction between parental substance abuse (SA) and analysis time, (ii) Schoenfeld residuals, and (iii) visual examination of Kaplan-Meier curves. In both of the age periods (infancy and ages 1–9), interaction effects between mother’s and father’s SA and analysis time were small in magnitude and not statistically significant (HRs between .67 and 1.01, all p-values > .05). Correlations between Schoenfeld residuals and analysis time did not show significant non-proportionality either (Rhos for correlation between analysis time and Schoenfeld residuals all max. ± .02, all p-values > .10). Further, Kaplan-Meier plots in infancy and ages 1–9 showed roughly parallel survival rates between those with and without parental SA throughout the follow-up period (Figs. S2 & S3). Thus, we concluded that the proportional hazards assumption of Cox regression was met for analyses in infancy and ages 1–9.


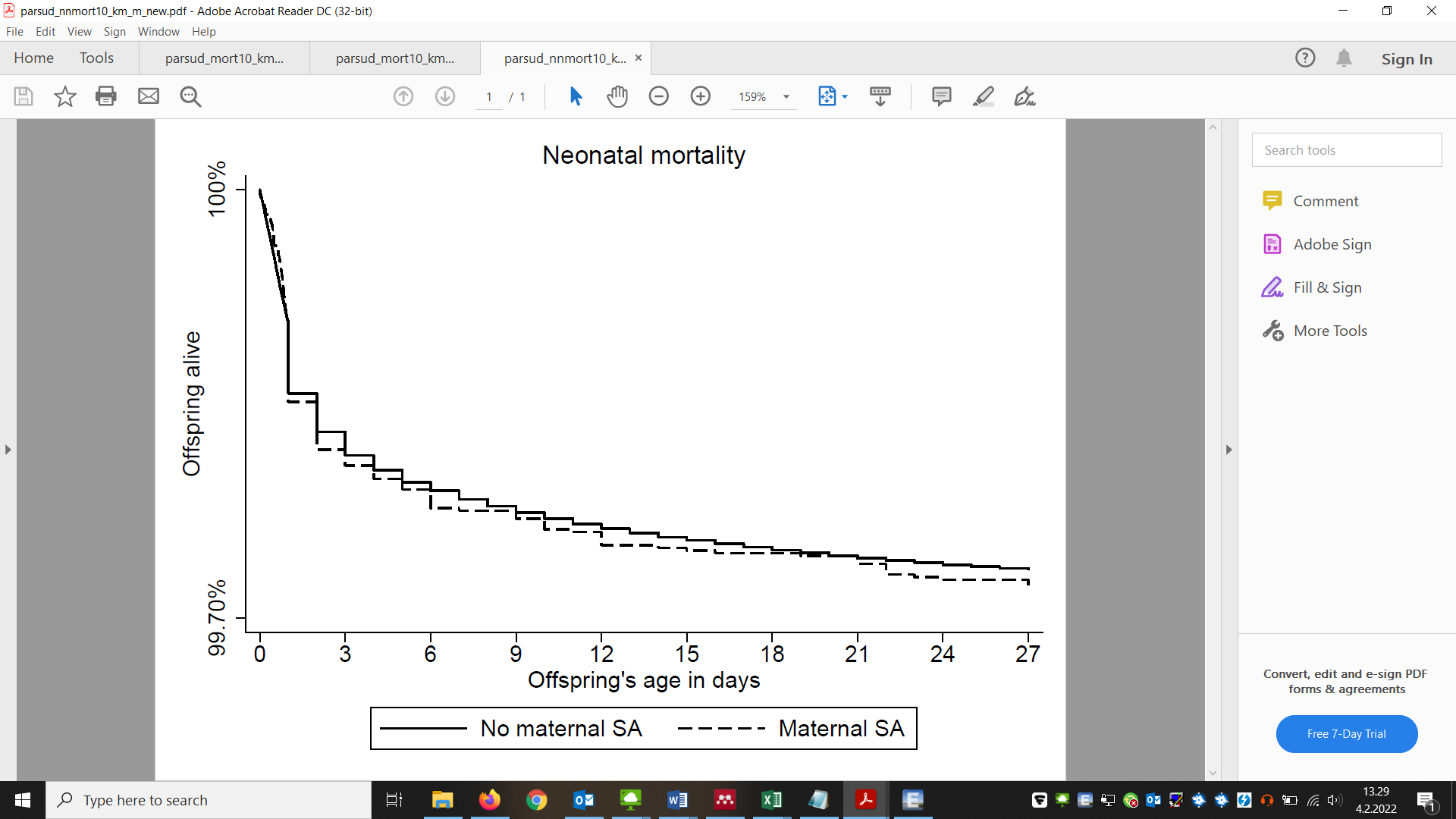

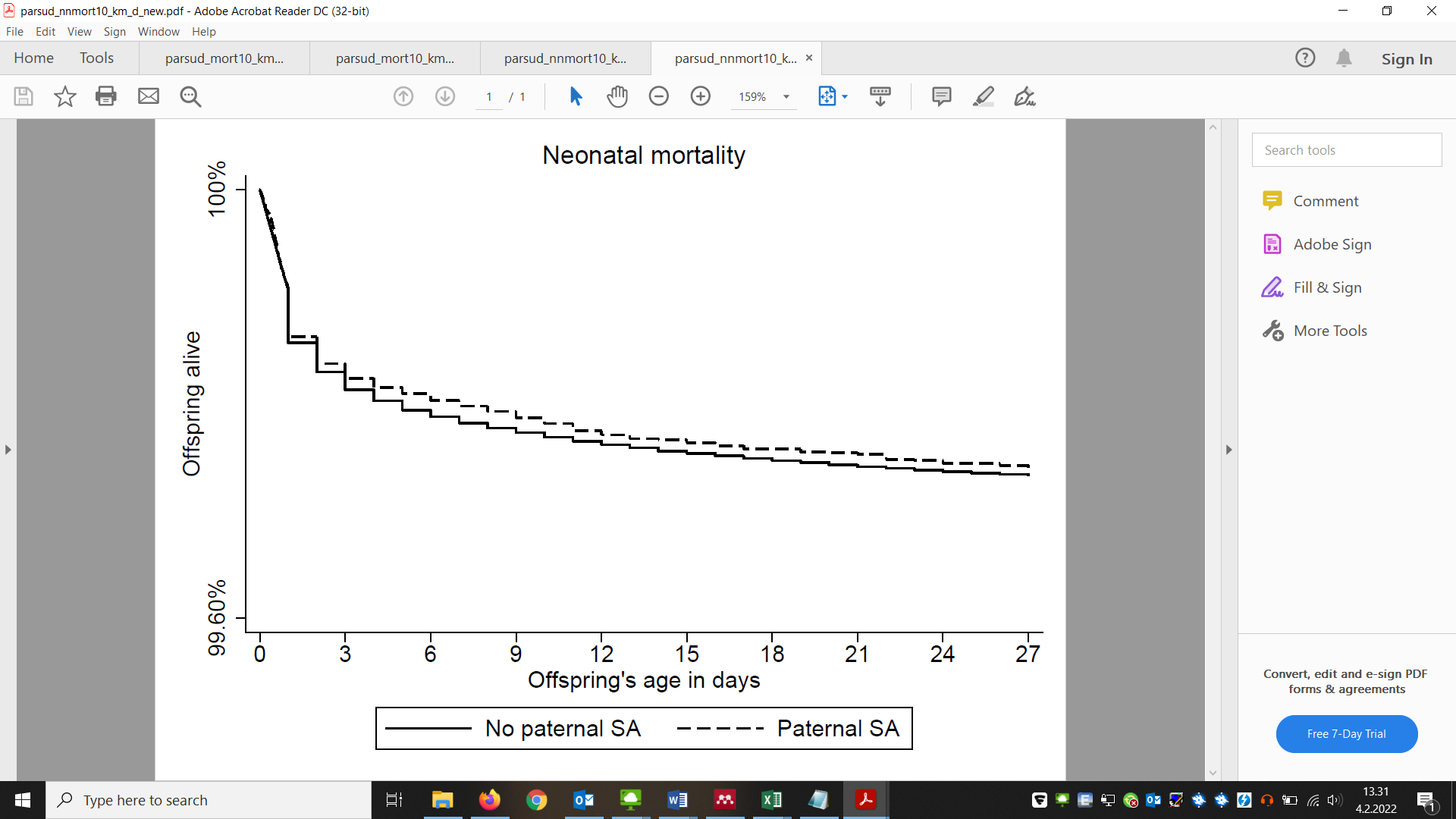


Fig. S1. Kaplan-Meier survival curves for offspring by mother’s and father’s SA in the neonatal period (first 27 days of life).


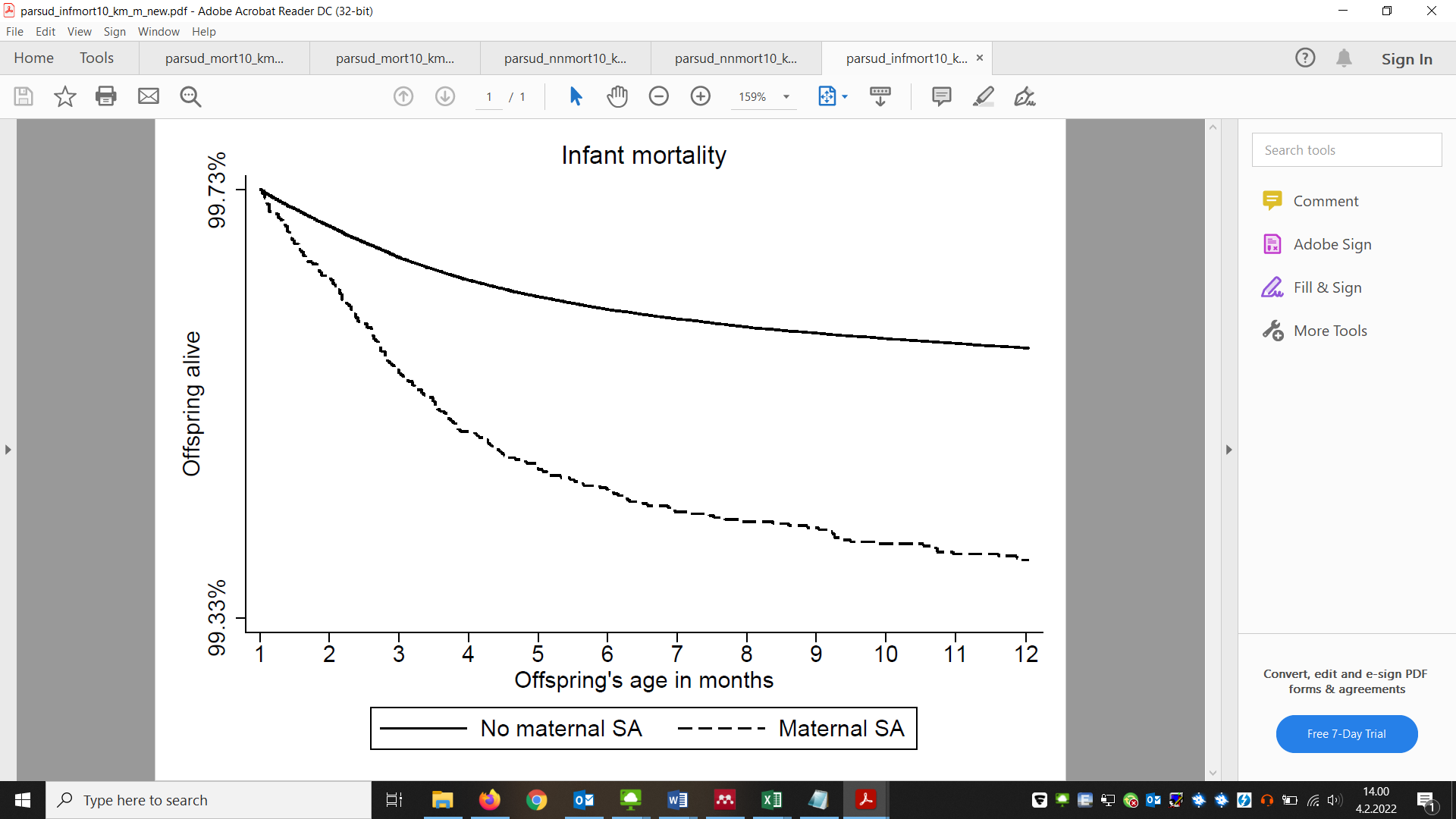

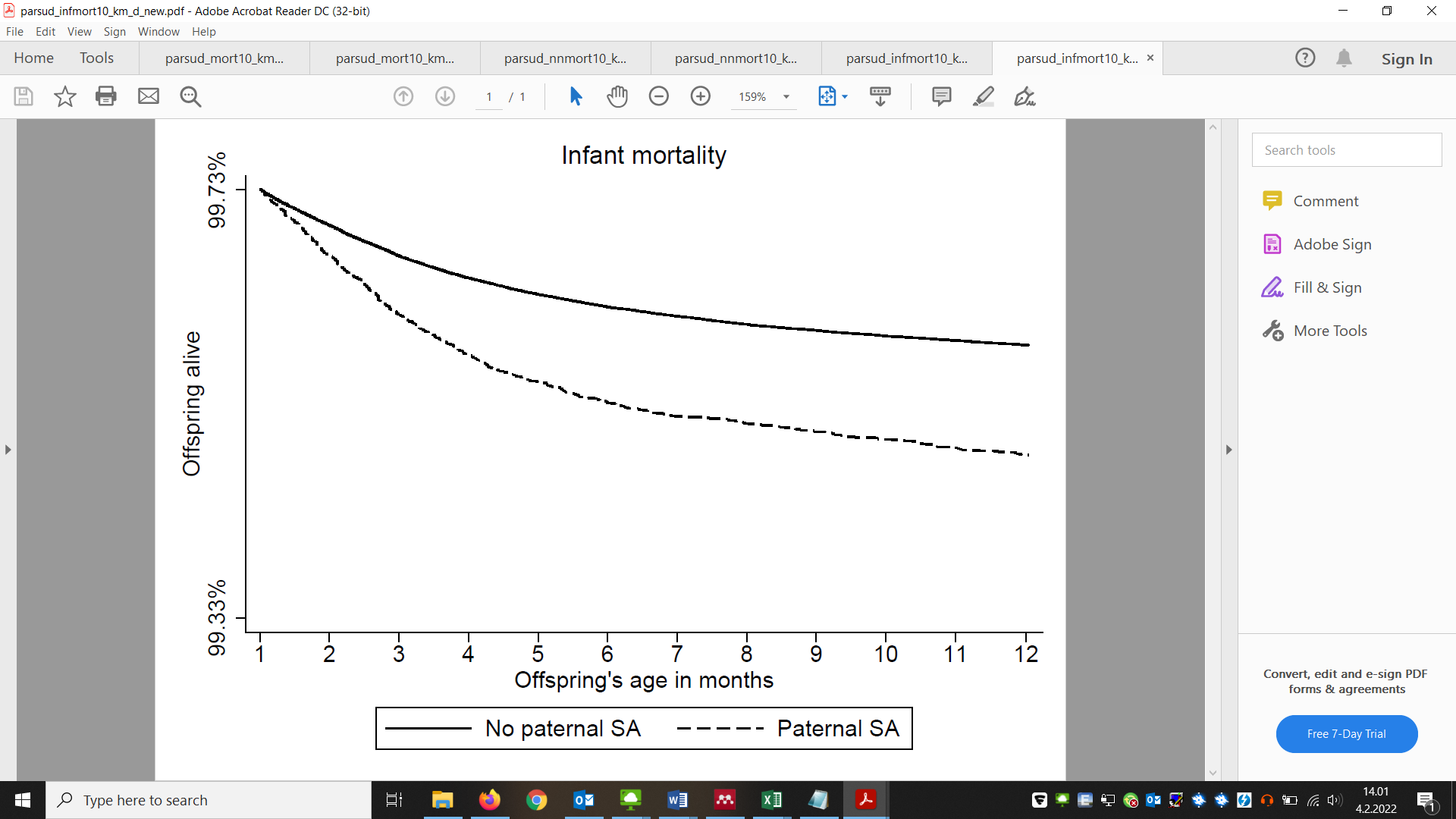


Fig. S2. Kaplan-Meier survival curves for offspring in infancy, by mother’s and father’s SA.


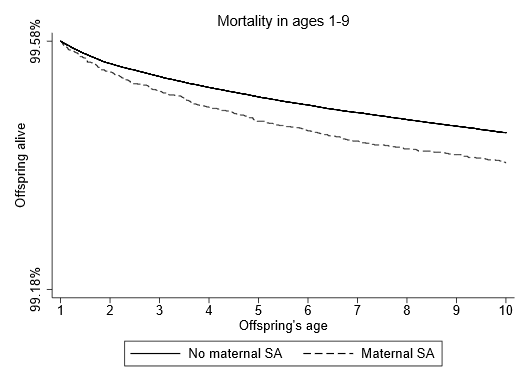

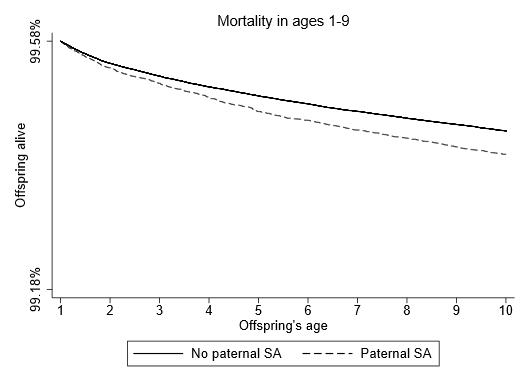


Fig. S3. Kaplan-Meier survival curves for offspring in ages 1–9, by mother’s and father’s SA.


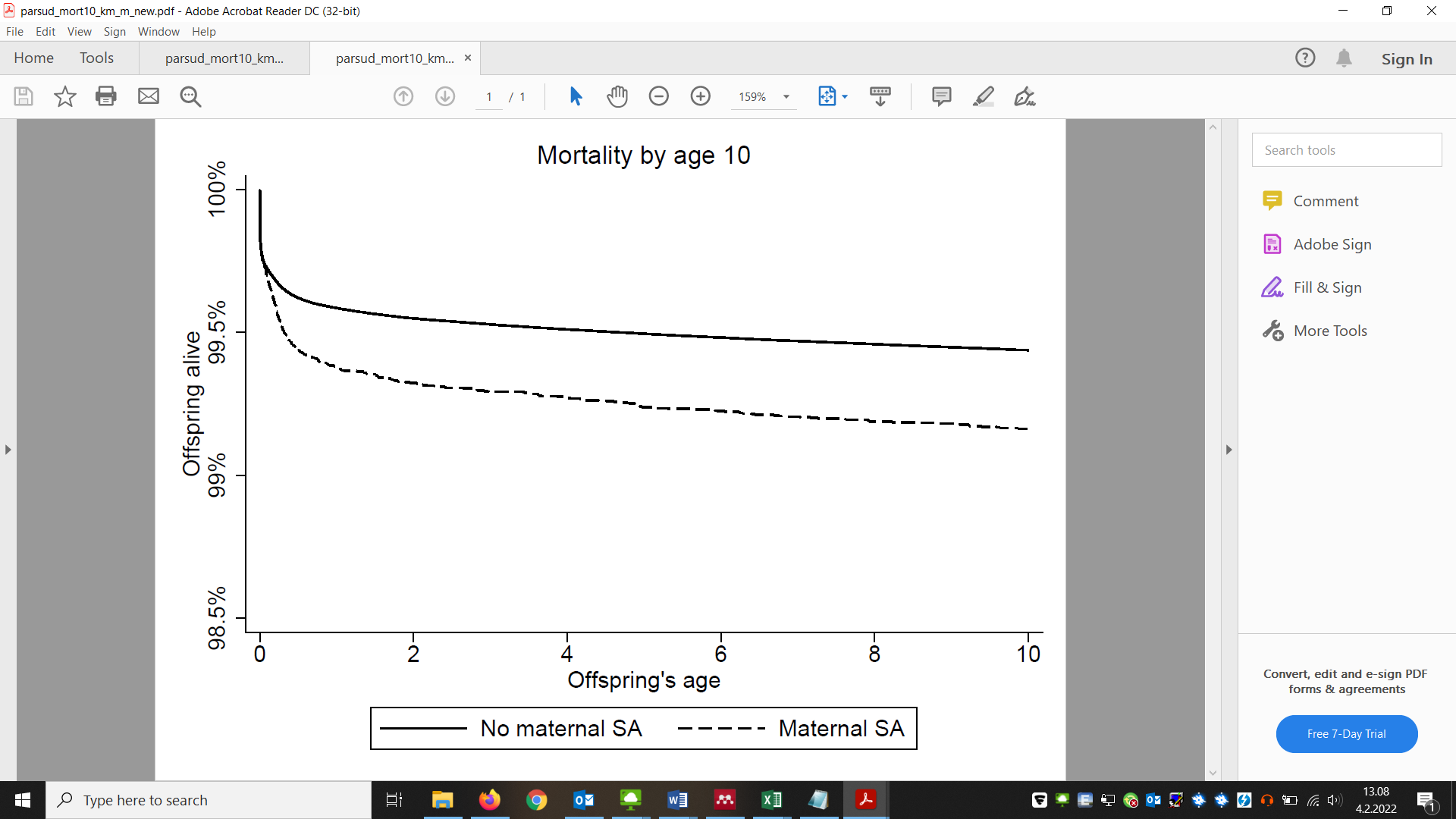

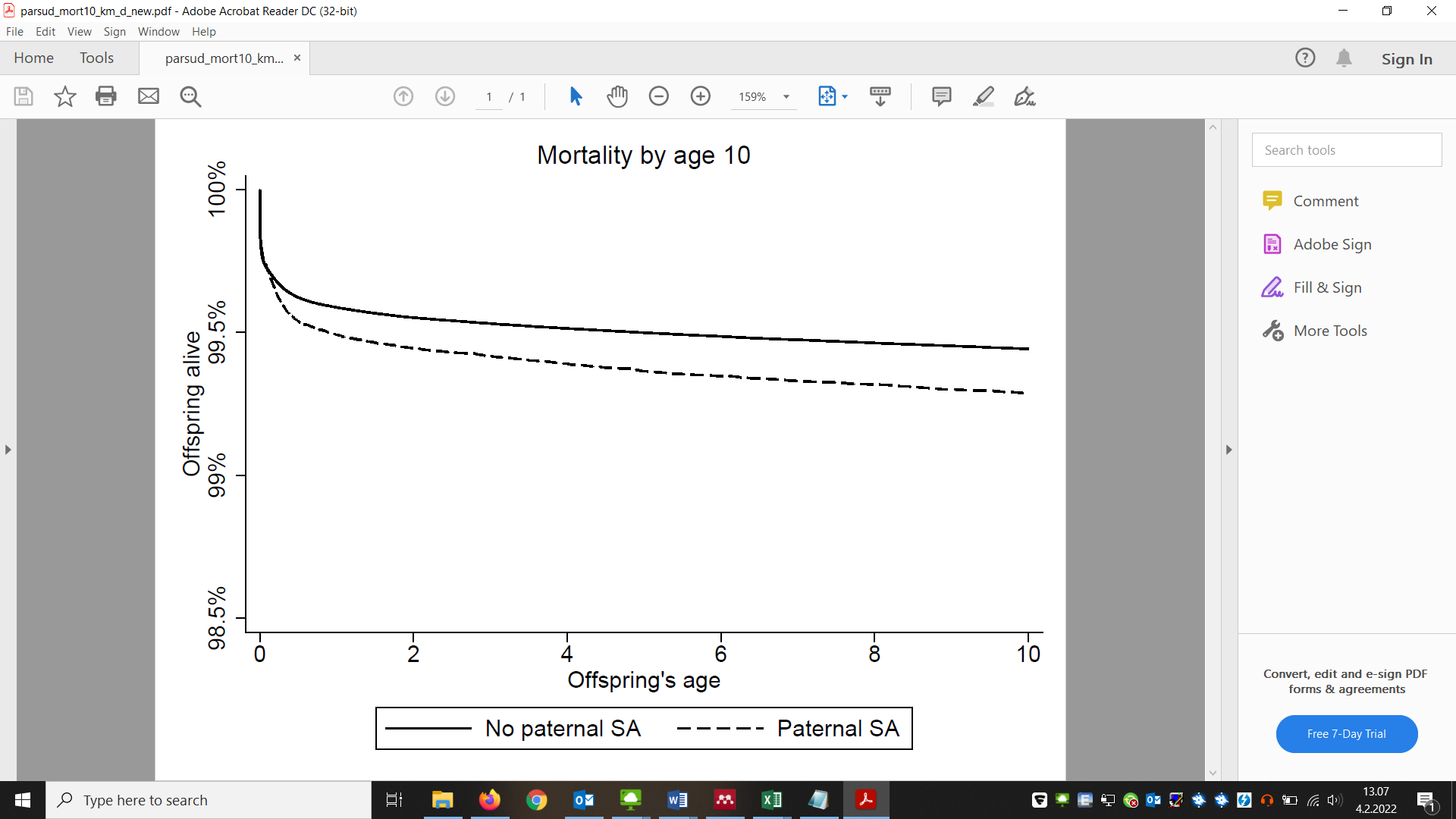


Fig. S4. Kaplan-Meier survival curves for offspring by mother’s and father’s SA through the follow-up period.


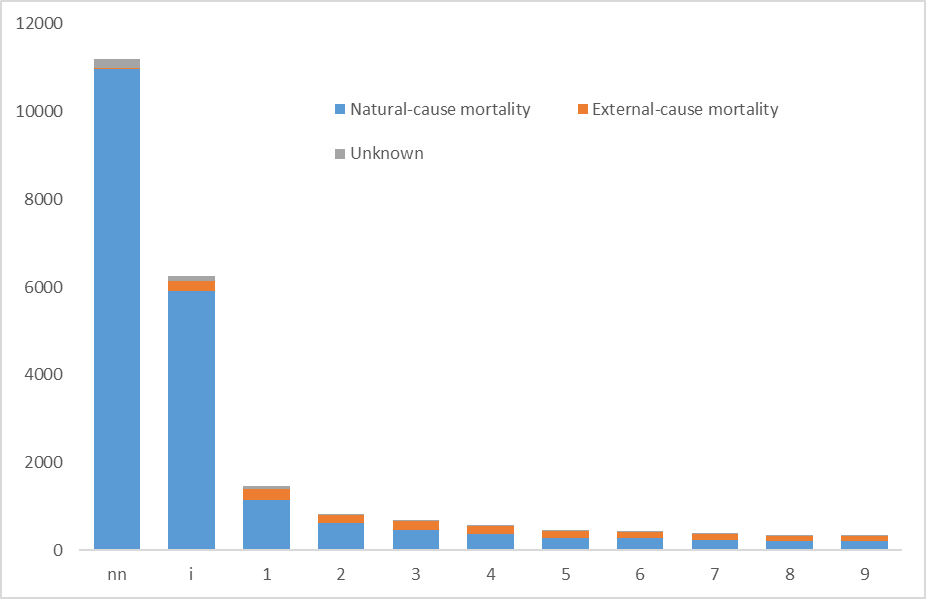


Fig. S5. All offspring deaths by age at death. Offspring age on the x-axis; nn = neonatal period (first 27 days); i = infancy (28 days – 12 months).

Fig. S6. Proportions of causes of death within age groups. Offspring age on the x-axis; nn = neonatal period (first 27 days); i = infancy (28 days–12 months). SIDS = sudden infant death syndrome.

Tables S3–S8 show results from Cox regressions predicting all-cause, natural-cause, and external-cause infant mortality and mortality in ages 1–9 by parental substance abuse and a set of other childhood risk factors available in registers. Missing information on parental education was coded as an additional category and included in the analyses (results not shown). Note that since neonatal mortality was not associated with parental SA in the non-adjusted models, results for adjusted models are not shown.

| Table S3. Infant all-cause mortality by parental substance abuse (SA) and covariates. | | | | |
| --- | --- | --- | --- | --- |
|  | Model 1 | | Model 2 | |
|  | HR (95% CI) | p | HR (95% CI) | p |
| Mother's SA | 2.23 (1.93, 2.59) | <.001 | 1.75 (1.48, 2.07) | <.001 |
| Father's SA | 1.53 (1.40, 1.67) | <.001 | 1.26 (1.14, 1.39) | <.001 |
| Severe mental disorder | | |  |  |
| Mother's | 1.43 (1.19, 1.73) | <.001 | 1.23 (1.00, 1.51) | .045 |
| Father's | 1.53 (1.23, 1.90) | <.001 | 1.22 (0.96, 1.54) | .109 |
| Education (ref. Highest) | | |  |  |
| Mother’s |  |  |  |  |
| Middle | 1.24 (1.17, 1.31) | <.001 | 1.15 (1.08, 1.23) | <.001 |
| Lowest | 1.59 (1.48, 1.72) | <.001 | 1.39 (1.27, 1.51) | <.001 |
| Father’s |  |  |  |  |
| Middle | 1.23 (1.16, 1.31) | <.001 | 1.07 (1.00, 1.15) | .048 |
| Lowest | 1.36 (1.27, 1.47) | <.001 | 1.07 (0.98, 1.16) | .139 |
| Father's income decile | 0.93 (0.92, 0.94) | <.001 | 0.95 (0.94, 0.96) | <.001 |
| Immigrant | |  |  |  |
| Mother | 1.20 (1.13, 1.29) | <.001 | 1.05 (0.96, 1.15) | .320 |
| Father | 1.18 (1.11, 1.26) | <.001 | 1.03 (0.94, 1.12) | .595 |
| Note. Both models control for offspring's sex and birth year, mother's age at offspring's birth and quadratic mother's age at offspring's birth. | | | | |
| Model 1 = Univariate models + basic covariates. | | | | |
| Model 2 = All predictors mutually adjusted + basic covariates. | | | | |

| Table S4. Infant natural-cause mortality by parental substance abuse (SA) and covariates. | | | | |
| --- | --- | --- | --- | --- |
|  | Model 1 | | Model 2 | |
|  | HR (95% CI) | p | HR (95% CI) | p |
| Mother's SA | 2.13 (1.82, 2.49) | <.001 | 1.71 (1.44, 2.04) | <.001 |
| Father's SA | 1.49 (1.36, 1.64) | <.001 | 1.24 (1.12, 1.38) | <.001 |
| Severe mental disorder | | |  |  |
| Mother's | 1.34 (1.10, 1.63) | .004 | 1.18 (0.96, 1.46) | .118 |
| Father's | 1.42 (1.13, 1.79) | .003 | 1.16 (0.90, 1.49) | .249 |
| Education (ref. Highest) | | |  |  |
| Mother |  |  |  |  |
| Middle | 1.24 (1.17, 1.32) | <.001 | 1.15 (1.07, 1.23) | <.001 |
| Lowest | 1.56 (1.45, 1.69) | <.001 | 1.36 (1.25, 1.49) | <.001 |
| Father |  |  |  |  |
| Middle | 1.24 (1.17, 1.33) | <.001 | 1.08 (1.01, 1.16) | .026 |
| Lowest | 1.37 (1.27, 1.47) | <.001 | 1.08 (0.99, 1.17) | .102 |
| Father's income decile | 0.93 (0.92, 0.94) | <.001 | 0.95 (0.94, 0.96) | <.001 |
| Immigrant | |  |  |  |
| Mother | 1.15 (1.08, 1.24) | <.001 | 1.03 (0.93, 1.13) | .586 |
| Father | 1.13 (1.06, 1.21) | <.001 | 1.00 (0.91, 1.10) | .952 |
| Note. Both models control for offspring's sex and birth year, mother's age at offspring's birth and quadratic mother's age at offspring's birth. | | | | |
| Model 1 = Univariate models + basic covariates. | | | | |
| Model 2 = All predictors mutually adjusted + basic covariates. | | | | |

| Table S5. Infant external-cause mortality by parental substance abuse (SA) and covariates. | | | | |
| --- | --- | --- | --- | --- |
|  | Model 1 | | Model 2 | |
|  | HR (95% CI) | p | HR (95% CI) | p |
| Mother's SA | 4.81 (2.78, 8.31) | <.001 | 2.85 (1.48, 5.49) | .002 |
| Father's SA | 2.15 (1.42, 3.24) | <.001 | 1.46 (0.91, 2.34) | .117 |
| Severe mental disorder | | |  |  |
| Mother's | 3.65 (1.94, 6.89) | <.001 | 2.55 (1.24, 5.25) | .011 |
| Father's | 4.21 (2.08, 8.53) | <.001 | 2.64 (1.16, 6.02) | .021 |
| Education (ref. Highest) | | |  |  |
| Mother |  |  |  |  |
| Middle | 1.19 (0.86, 1.64) | .302 | 1.44 (0.99, 2.11) | .058 |
| Lowest | 1.85 (1.25, 2.74) | .002 | 1.93 (1.20, 3.09) | .006 |
| Father |  |  |  |  |
| Middle | 0.87 (0.63, 1.20) | .401 | 0.74 (0.51, 1.08) | .124 |
| Lowest | 1.08 (0.74, 1.58) | .685 | 0.72 (0.46, 1.13) | .147 |
| Father's income decile | 0.87 (0.82, 0.93) | <.001 | 0.92 (0.86, 0.99) | .021 |
| Immigrant | |  |  |  |
| Mother | 2.07 (1.52, 2.81) | <.001 | 1.29 (0.82, 2.03) | .271 |
| Father | 1.84 (1.35, 2.51) | <.001 | 1.26 (0.80, 1.97) | .322 |
| Note. Both models control for offspring's sex and birth year, mother's age at offspring's birth and quadratic mother's age at offspring's birth. | | | | |
| Model 1 = Univariate models + basic covariates. | | | | |
| Model 2 = All predictors mutually adjusted + basic covariates. | | | | |

| Table S6. All-cause mortality in ages 1–9 by parental substance abuse (SA) and covariates. | | | | |
| --- | --- | --- | --- | --- |
|  | Model 1 | | Model 2 | |
|  | HR (95% CI) | p | HR (95% CI) | p |
| Mother's SA | 1.45 (1.20, 1.75) | <.001 | 1.17 (0.94, 1.46) | .155 |
| Father's SA | 1.31 (1.18, 1.45) | <.001 | 1.09 (0.97, 1.22) | .157 |
| Severe mental disorder | | |  |  |
| Mother's | 1.27 (1.03, 1.56) | .026 | 1.21 (0.97, 1.51) | .089 |
| Father's | 1.14 (0.88, 1.48) | .333 | 1.02 (0.77, 1.35) | .903 |
| Education (ref. Highest) | | |  |  |
| Mother |  |  |  |  |
| Middle | 1.19 (1.12, 1.27) | <.001 | 1.09 (1.01, 1.16) | .018 |
| Lowest | 1.57 (1.45, 1.70) | <.001 | 1.31 (1.19, 1.44) | <.001 |
| Father |  |  |  |  |
| Middle | 1.21 (1.13, 1.29) | <.001 | 1.06 (0.99, 1.15) | .102 |
| Lowest | 1.38 (1.27, 1.49) | <.001 | 1.13 (1.03, 1.23) | .009 |
| Father's income decile | 0.93 (0.92, 0.94) | <.001 | 0.95 (0.94, 0.96) | <.001 |
| Immigrant | |  |  |  |
| Mother | 1.31 (1.22, 1.41) | <.001 | 1.19 (1.08, 1.31) | <.001 |
| Father | 1.24 (1.15, 1.33) | <.001 | 0.98 (0.89, 1.09) | .754 |
| Note. Both models control for offspring's sex and birth year, mother's age at offspring's birth and quadratic mother's age at offspring's birth. | | | | |
| Model 1 = Univariate models + basic covariates. | | | | |
| Model 2 = All predictors mutually adjusted + basic covariates. | | | | |

| Table S7. Natural-cause mortality in ages 1–9 by parental substance abuse (SA) and covariates. | | | | |
| --- | --- | --- | --- | --- |
|  | Model 1 | | Model 2 | |
|  | HR (95% CI) | p | HR (95% CI) | p |
| Mother's SA | 1.08 (0.83, 1.41) | .571 | 1.01 (0.75, 1.35) | .973 |
| Father's SA | 1.08 (0.95, 1.24) | .240 | 0.96 (0.83, 1.11) | .576 |
| Severe mental disorder | | |  |  |
| Mother's | 1.03 (0.78, 1.36) | .857 | 1.02 (0.76, 1.37) | .879 |
| Father's | 1.20 (0.88, 1.64) | .239 | 1.06 (0.76, 1.48) | .734 |
| Education (ref. Highest) | | |  |  |
| Mother |  |  |  |  |
| Middle | 1.10 (1.02, 1.18) | .010 | 1.03 (0.95, 1.11) | .513 |
| Lowest | 1.40 (1.28, 1.54) | <.001 | 1.23 (1.10, 1.37) | <.001 |
| Father |  |  |  |  |
| Middle | 1.16 (1.08, 1.26) | <.001 | 1.07 (0.98, 1.16) | .139 |
| Lowest | 1.27 (1.15, 1.39) | <.001 | 1.10 (0.99, 1.22) | .080 |
| Father's income decile | 0.95 (0.94, 0.96) | <.001 | 0.96 (0.95, 0.98) | <.001 |
| Immigrant | |  |  |  |
| Mother | 1.21 (1.11, 1.31) | <.001 | 1.15 (1.03, 1.29) | .017 |
| Father | 1.13 (1.04, 1.24) | .004 | 0.97 (0.86, 1.09) | .615 |
| Note. Both models control for offspring's sex and birth year, mother's age at offspring's birth and quadratic mother's age at offspring's birth. | | | | |
| Model 1 = Univariate models + basic covariates. | | | | |
| Model 2 = All predictors mutually adjusted + basic covariates. | | | | |

| Table S8. External-cause mortality in ages 1–9 by parental substance abuse (SA) and covariates. | | | | |
| --- | --- | --- | --- | --- |
|  | Model 1 | | Model 2 | |
|  | HR (95% CI) | p | HR (95% CI) | p |
| Mother's SA | 2.51 (1.90, 3.33) | <.001 | 1.53 (1.10, 2.15) | .013 |
| Father's SA | 1.90 (1.61, 2.24) | <.001 | 1.37 (1.14, 1.66) | .001 |
| Severe mental disorder | | |  |  |
| Mother's | 1.91 (1.38, 2.64) | <.001 | 1.66 (1.17, 2.37) | .005 |
| Father's | 0.87 (0.49, 1.54) | .634 | 0.80 (0.45, 1.42) | .454 |
| Education (ref. Highest) | | |  |  |
| Mother |  |  |  |  |
| Middle | 1.49 (1.31, 1.69) | <.001 | 1.28 (1.11, 1.48) | .001 |
| Lowest | 2.10 (1.80, 2.45) | <.001 | 1.57 (1.31, 1.87) | <.001 |
| Father |  |  |  |  |
| Middle | 1.44 (1.26, 1.66) | <.001 | 1.13 (0.97, 1.32) | .123 |
| Lowest | 1.83 (1.57, 2.13) | <.001 | 1.28 (1.07, 1.53) | .006 |
| Father's income decile | 0.89 (0.87, 0.91) | <.001 | 0.92 (0.90, 0.95) | <.001 |
| Immigrant | |  |  |  |
| Mother | 1.43 (1.25, 1.64) | <.001 | 1.24 (1.03, 1.49) | .021 |
| Father | 1.30 (1.14, 1.49) | <.001 | 0.88 (0.73, 1.07) | .192 |
| Note. Both models control for offspring's sex and birth year, mother's age at offspring's birth and quadratic mother's age at offspring's birth. | | | | |
| Model 1 = Univariate models + basic covariates. | | | | |
| Model 2 = All predictors mutually adjusted + basic covariates. | | | | |

| Table S9. Results from logistic regressions predicting sudden infant death syndrome by parental substance abuse (SA) and covariates. | | | | |
| --- | --- | --- | --- | --- |
|  | Model 1 | | Model 2 | |
|  | OR (95% CI) | p | OR (95% CI) | p |
| Mother's SA | 3.77 (3.07, 4.64) | <.001 | 2.11 (1.70, 2.62) | <.001 |
| Father's SA | 2.26 (1.97, 2.60) | <.001 | 1.59 (1.37, 1.83) | <.001 |
| SDP | 3.19 (2.86, 3.56) | <.001 | 2.81 (2.51, 3.15) | <.001 |
| Preterm birth | |  |  |  |
| Late (34<37 weeks) | 2.30 (1.96, 2.70) | <.001 | 2.14 (1.82, 2.51) | <.001 |
| Early (<34 weeks) | 4.27 (3.50, 5.20) | <.001 | 3.94 (3.24, 4.80) | <.001 |
| Note. Both models control for offspring's sex and birth year, mother's age at offspring's birth and quadratic mother's age at offspring's birth. | | | | |
| Model 1 = Univariate models + basic covariates. | | | |  |
| Models 2 = Multivariate model with all predictors mutually adjusted + basic covariates. | | | | |
| SDP = Maternal smoking during pregnancy | | | |  |

| Table S10. Results from analyses predicting all-cause and cause-specific mortality in different ages by offspring's sex and an interaction term between offspring sex and parental SA. | | | | | | |
| --- | --- | --- | --- | --- | --- | --- |
|  | Neonatal mortality | | Infant mortality | | Mortality in ages 1–9 | |
|  | OR (95%CI) | p* | HR (95% CI) | p* | HR (95% CI) | p* |
| **All-cause mortality** | |  |  |  |  |  |
| Boy (ref. Girl) | 1.27 (1.22, 1.32) |  | 1.21 (1.15, 1.27) |  | 1.26 (1.19, 1.33) |  |
| Mother's SA in girls | 1.12 (0.88, 1.43) |  | 2.27 (1.82, 2.83) |  | 1.37 (1.01, 1.85) |  |
| Mother's SA in boys | 1.06 (0.86, 1.32) | .759 | 2.20 (1.81, 2.68) | .839 | 1.51 (1.18, 1.93) | .632 |
| Father's SA in girls | 0.94 (0.83, 1.07) |  | 1.48 (1.29, 1.69) |  | 1.18 (1.00, 1.39) |  |
| Father's SA in boys | 0.94 (0.84, 1.05) | .981 | 1.57 (1.39, 1.76) | .523 | 1.40 (1.23, 1.60) | .102 |
| **Natural-cause mortality** | | |  |  |  |  |
| Boy (ref. Girl) | 1.27 (1.22, 1.32) |  | 1.22 (1.15, 1.28) |  | 1.15 (1.07, 1.22) |  |
| Mother's SA in girls | 1.12 (0.87, 1.43) |  | 2.13 (1.68, 2.69) |  | 0.88 (0.56, 1.36) |  |
| Mother's SA in boys | 1.09 (0.88, 1.35) | .893 | 2.13 (1.74, 2.62) | .994 | 1.25 (0.89, 1.74) | .211 |
| Father's SA in girls | 0.93 (0.82, 1.06) |  | 1.42 (1.23, 1.64) |  | 1.01 (0.83, 1.24) |  |
| Father's SA in boys | 0.92 (0.82, 1.03) | .882 | 1.54 (1.36, 1.74) | .414 | 1.14 (0.96, 1.36) | .393 |
| Infectious disease | |  |  |  |  |  |
| Boy (ref. Girl) | 1.29 (1.04, 1.60) |  | 1.26 (1.07, 1.49) |  | 1.14 (0.96, 1.36) |  |
| Mother's SA in girls | 1.73 (0.55, 5.42) |  | 2.46 (1.26, 4.79) |  | 0.30 (0.04, 2.12) |  |
| Mother's SA in boys | 1.25 (0.40, 3.90) | .692 | 2.22 (1.21, 4.05) | .821 | 2.18 (1.12, 4.25) | .059 |
| Father's SA in girls | 0.71 (0.31, 1.61) |  | 1.76 (1.19, 2.62) |  | 1.74 (1.14, 2.66) |  |
| Father's SA in boys | 0.88 (0.46, 1.66) | .692 | 2.12 (1.54, 2.92) | .471 | 1.31 (0.85, 2.01) | .352 |
| Neoplasms | |  |  |  |  |  |
| Boy (ref. Girl) | 1.09 (0.65, 1.82) |  | 0.85 (0.60, 1.20) |  | 1.17 (1.04, 1.31) |  |
| Mother's SA in girls | a |  | a |  | 1.30 (0.67, 2.52) |  |
| Mother's SA in boys | a |  | a |  | 0.92 (0.46, 1.86) | .483 |
| Father's SA in girls | 2.35 (0.70, 7.87) |  | 0.52 (0.13, 2.13) |  | 0.83 (0.55, 1.24) |  |
| Father's SA in boys | 0.62 (0.08, 4.60) | .264 | 1.20 (0.43, 3.32) | .346 | 1.03 (0.74, 1.43) | .401 |
| Congenital malformations | | |  |  |  |  |
| Boy (ref. Girl) | 1.19 (1.12, 1.26) |  | 1.01 (0.93, 1.11) |  | 1.09 (0.96, 1.24) |  |
| Mother's SA in girls | 0.61 (0.35, 1.05) |  | 1.40 (0.88, 2.24) |  | 0.72 (0.27, 1.93) |  |
| Mother's SA in boys | 0.84 (0.56, 1.27) | .347 | 0.72 (0.38, 1.34) | .090 | 1.23 (0.61, 2.47) | .385 |
| Father's SA in girls | 0.83 (0.66, 1.05) |  | 0.96 (0.73, 1.27) |  | 0.79 (0.50, 1.24) |  |
| Father's SA in boys | 0.80 (0.65, 0.98) | .794 | 0.90 (0.69, 1.18) | .732 | 1.25 (0.89, 1.76) | .107 |
| **External-cause mortality** | | |  |  |  |  |
| Boy (ref. Girl) | a |  | 1.11 (0.84, 1.46) |  | 1.65 (1.48, 1.84) |  |
| Mother's SA in girls | a |  | 4.51 (1.97, 10.34) | | 3.14 (2.07, 4.77) |  |
| Mother's SA in boys | a |  | 5.06 (2.46, 10.41) | .838 | 2.16 (1.48, 3.15) | .192 |
| Father's SA in girls | a |  | 2.08 (1.13, 3.82) |  | 1.77 (1.35, 2.34) |  |
| Father's SA in boys | a |  | 2.21 (1.28, 3.82) | .881 | 1.97 (1.61, 2.42) | .539 |
| Accidents | a |  |  |  |  |  |
| Boy (ref. Girl) | a |  | 1.08 (0.79, 1.48) |  | 1.72 (1.54, 1.93) |  |
| Mother's SA in girls | a |  | 3.05 (0.96, 9.70) |  | 2.99 (1.89, 4.74) |  |
| Mother's SA in boys | a |  | 5.28 (2.29, 12.14) | .449 | 1.96 (1.29, 2.97) | .179 |
| Father's SA in girls | a |  | 2.37 (1.21, 4.64) |  | 1.72 (1.27, 2.32) |  |
| Father's SA in boys | a |  | 2.67 (1.47, 4.84) | .796 | 1.95 (1.58, 2.42) | .487 |
| Homicides | a |  |  |  |  |  |
| Boy (ref. Girl) | a |  | 0.95 (0.45, 1.99) |  | 1.13 (0.77, 1.66) |  |
| Mother's SA in girls | a |  | a |  | 6.12 (2.19, 17.07) |  |
| Mother's SA in boys | a |  | 4.29 (0.56, 33.17) | a | 5.05 (1.82, 13.98) | .793 |
| Father's SA in girls | a |  | a |  | 1.71 (0.67, 4.33) |  |
| Father's SA in boys | a |  | 2.20 (0.48, 9.98) | a | 1.74 (0.74, 4.07) | .977 |
| Note. Main effect of sex from the model with maternal SA. p*-value for the interaction term between sex and parental SA, i.e., indicates whether the association between parental SA and offspring mortality is different in boys and girls. ^a^Too few cases for model estimation. | | | | | | |


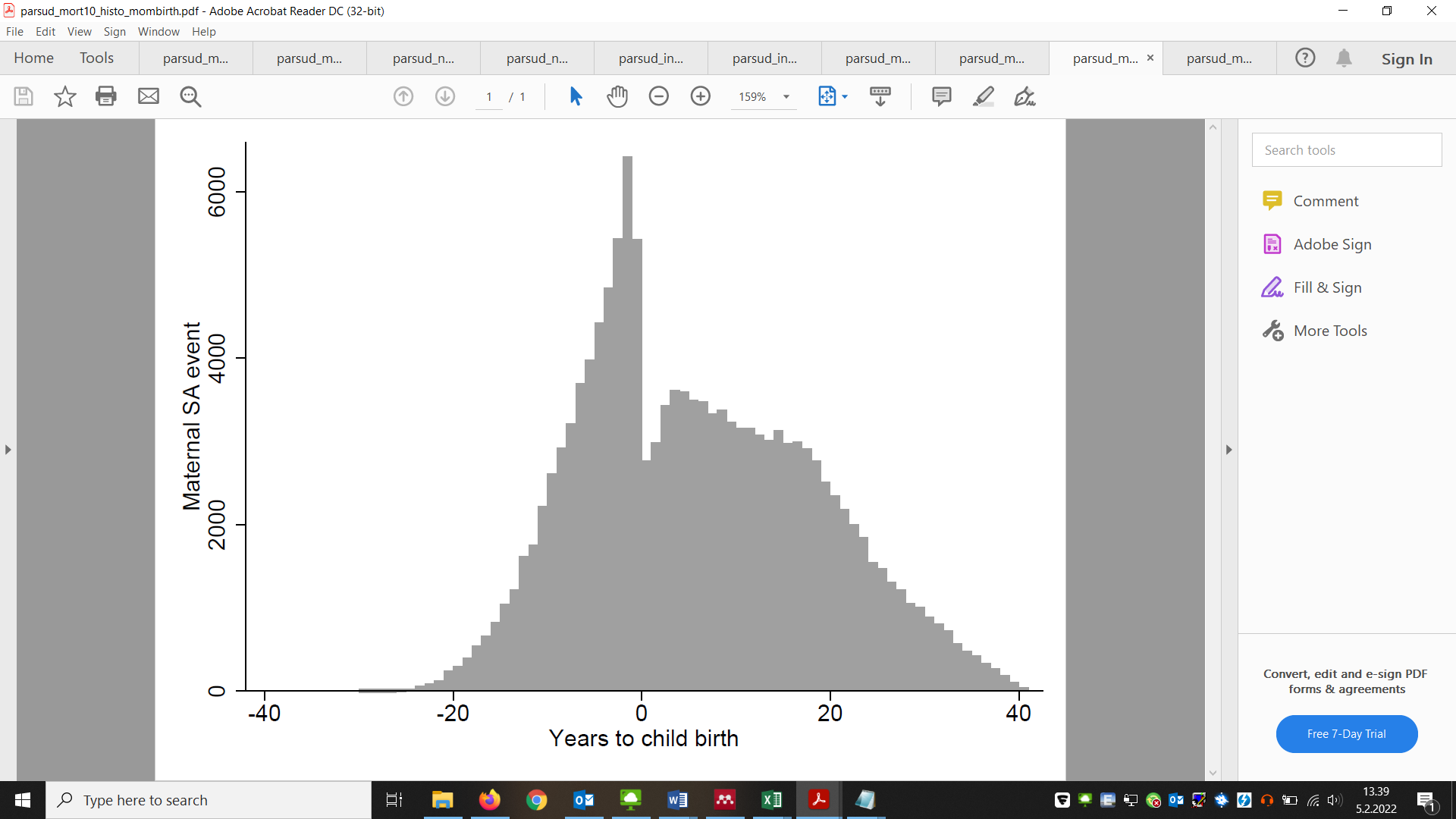

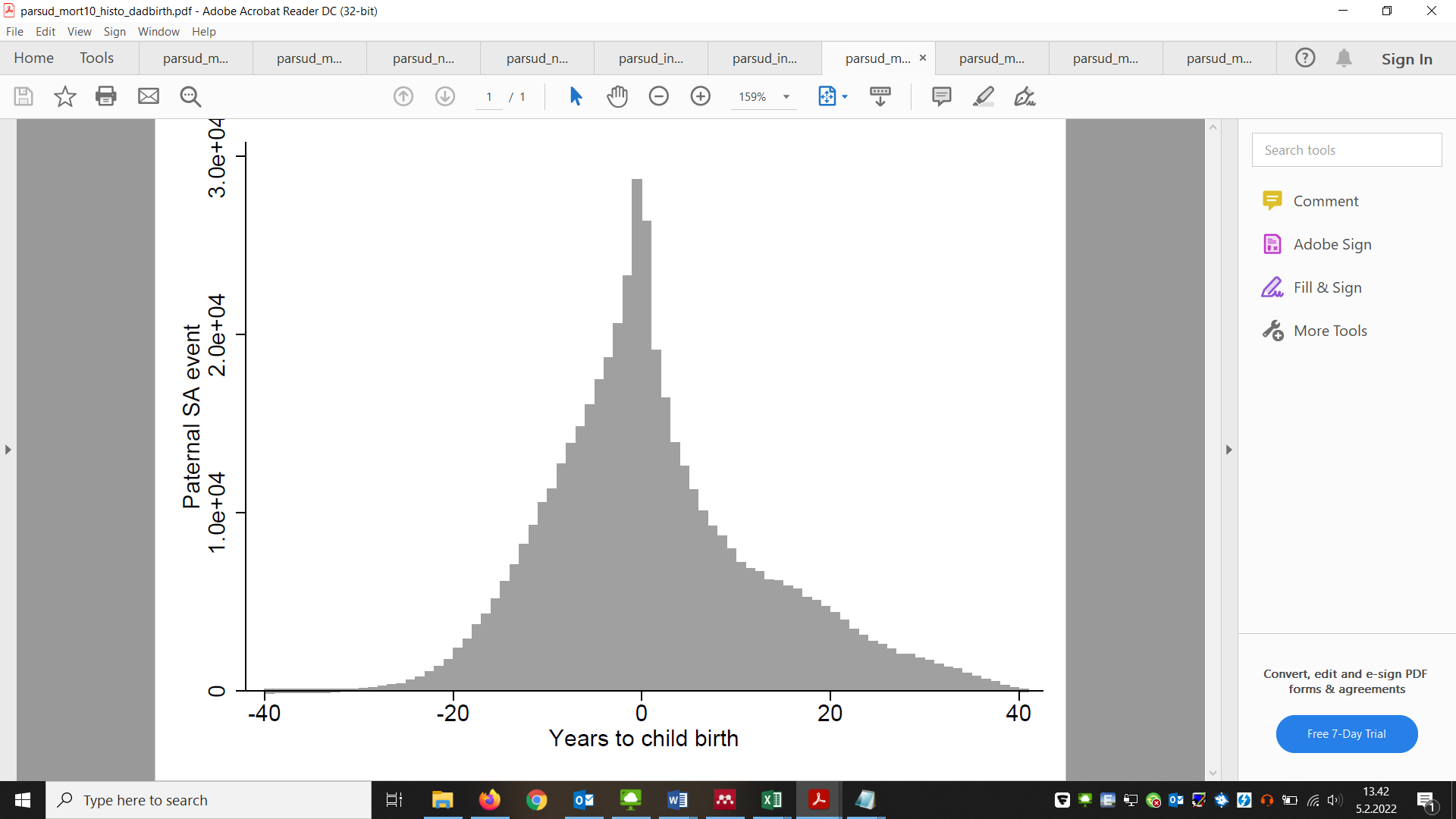


Fig. S7. Histograms showing the frequency of parental SA events in registers relative to focal child birth. Only the shortest interval between child birth and parental SA event is shown for each child.


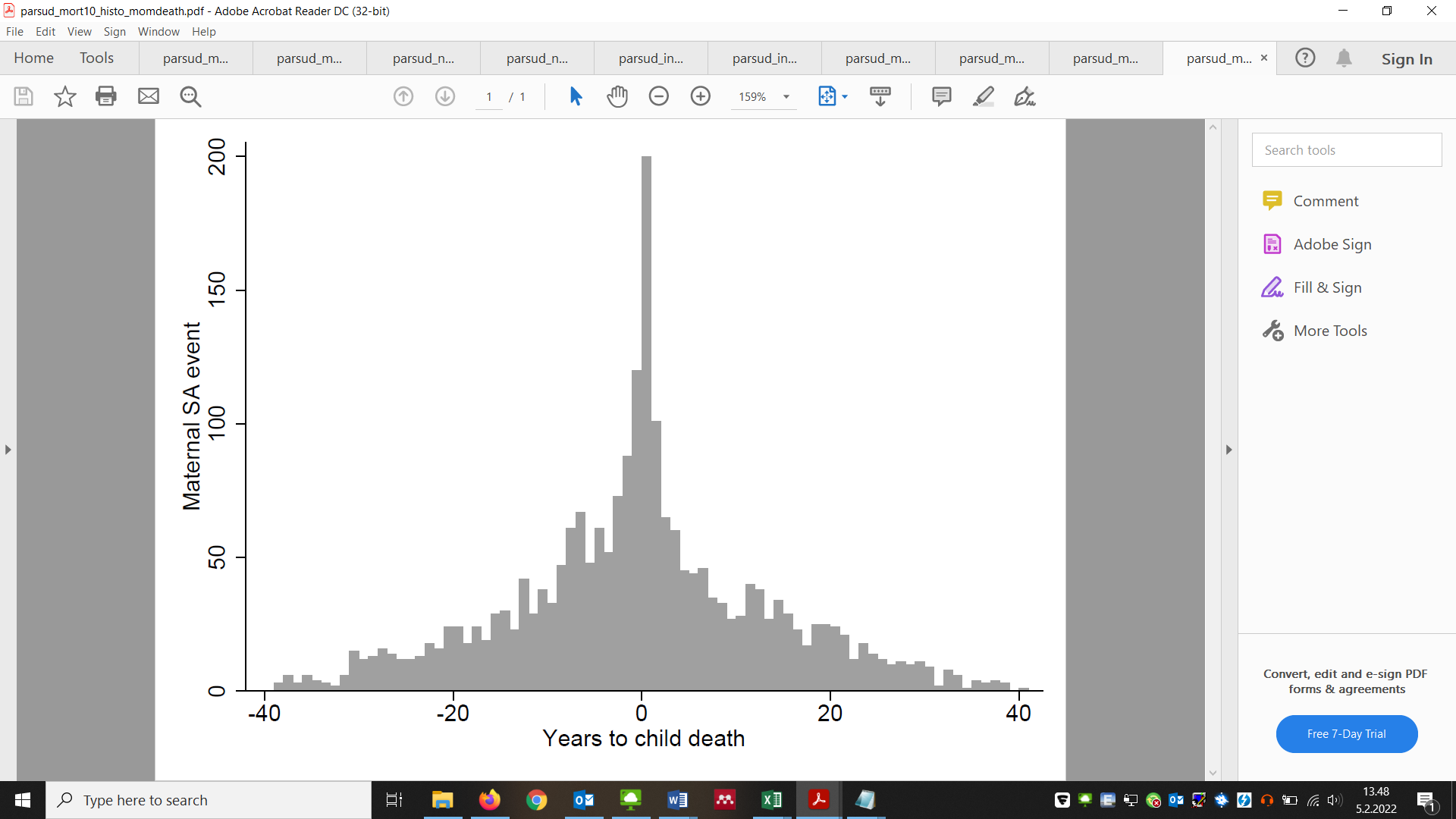

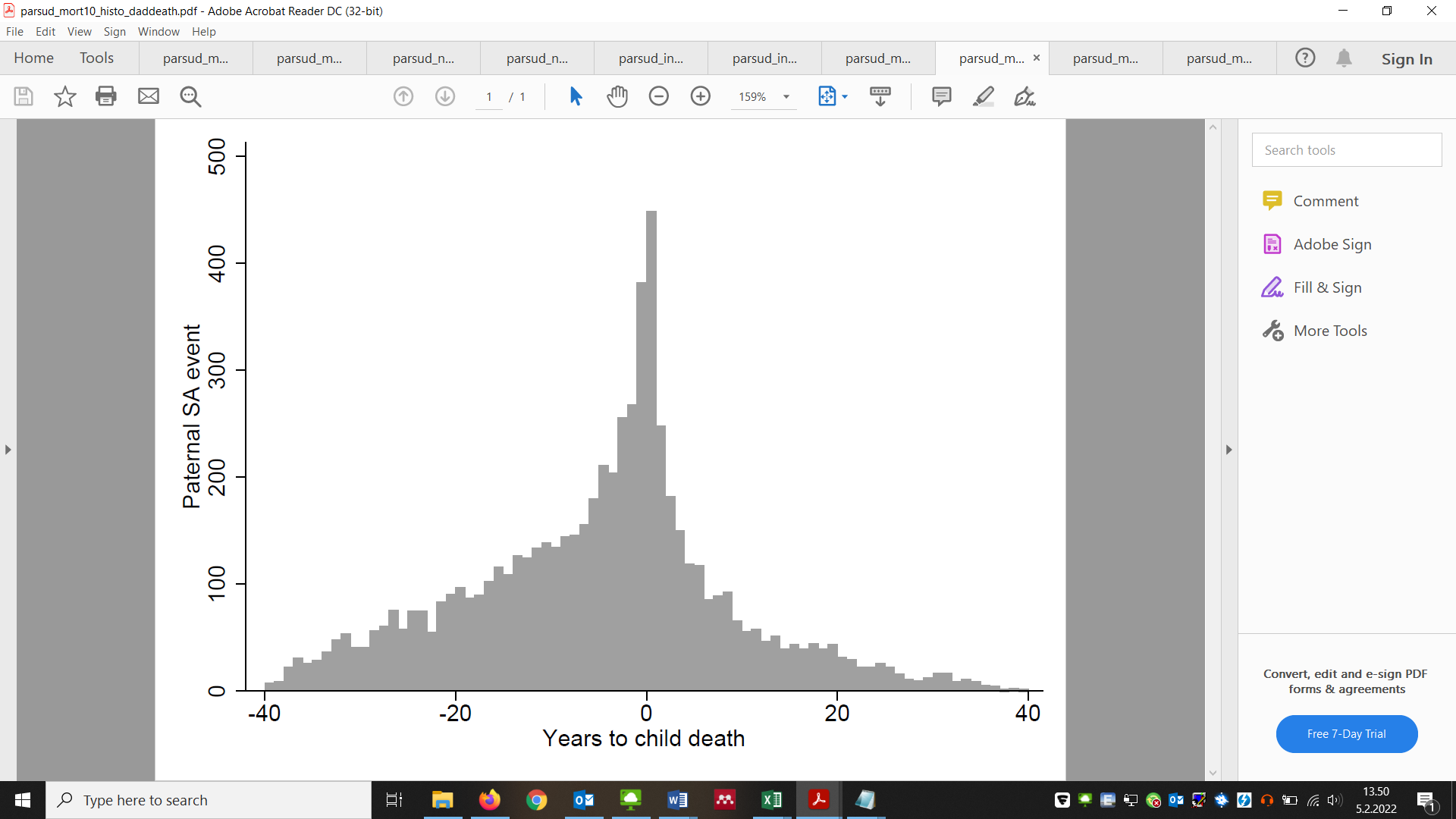


Fig. S8. Histograms showing the frequency of parental SA events in registers relative to focal child’s death. Only the shortest interval between child death and parental SA event is shown for each child.

| Table S11. Offspring mortality by time-varying parental substance abuse (SA). | | | | | | |
| --- | --- | --- | --- | --- | --- | --- |
|  | Neonatal mortality | | Infant mortality | | Mortality in ages 1–9 | |
|  | HR (95% CI) | p | HR (95% CI) | p | HR (95% CI) | p |
| All-cause mortality | |  |  |  |  |  |
| Mother's SA | 1.07 (0.89, 1.27) | .482 | 1.97 (1.67, 2.33) | <.001 | 1.18 (0.95, 1.46) | .137 |
| Father's SA | 0.94 (0.86, 1.02) | .113 | 1.58 (1.45, 1.71) | <.001 | 1.22 (1.11, 1.34) | <.001 |
| Natural-cause mortality | |  |  |  |  |  |
| Mother's SA | 1.07 (0.89, 1.28) | .453 | 1.96 (1.65, 2.33) | <.001 | 1.03 (0.78, 1.36) | .834 |
| Father's SA | 0.93 (0.86, 1.02) | .108 | 1.57 (1.44, 1.71) | <.001 | 1.01 (0.90, 1.14) | .829 |
| External-cause mortality | |  |  |  |  |  |
| Mother's SA | a |  | 2.56 (1.13, 5.80) | .024 | 1.70 (1.19, 2.41) | .003 |
| Father's SA | 1.16 (0.27, 4.94) | .843 | 1.85 (1.21, 2.82) | .004 | 1.79 (1.53, 2.09) | <.001 |
| Note. All models control for offspring's sex and birth year, mother's age at offspring's birth and quadratic mother's age at offspring's birth. ^a^Too few cases for model estimation. | | | | | | |

| Table S12. All-cause and cause-specific mortality in infancy by lifetime parental substance abuse (SA). | | | | |
| --- | --- | --- | --- | --- |
|  | Model 1 |  | Model 2 |  |
|  | HR (95% CI) | p | HR (95% CI) | p |
| **All-cause mortality** | |  |  |  |
| Mother's SA | 1.87 (1.68, 2.07) | <.001 | 1.63 (1.45, 1.82) | <.001 |
| Father's SA | 1.40 (1.31, 1.50) | <.001 | 1.20 (1.11, 1.29) | <.001 |
| **Natural-cause mortality** | | |  |  |
| Mother's SA | 1.82 (1.63, 2.02) | <.001 | 1.61 (1.43, 1.81) | <.001 |
| Father's SA | 1.39 (1.29, 1.49) | <.001 | 1.19 (1.10, 1.29) | <.001 |
| Infectious diseases | |  |  |  |
| Mother's SA | 2.05 (1.51, 2.79) | <.001 | 1.72 (1.22, 2.43) | .002 |
| Father's SA | 1.53 (1.24, 1.88) | <.001 | 1.29 (1.03, 1.63) | .028 |
| Neoplasms | |  |  |  |
| Mother's SA | 0.92 (0.34, 2.51) | .876 | 1.21 (0.44, 3.34) | .708 |
| Father's SA | 1.12 (0.67, 1.88) | .657 | 0.93 (0.51, 1.69) | .813 |
| Congenital malformations | | |  |  |
| Mother's SA | 1.17 (0.94, 1.46) | .160 | 1.21 (0.44, 3.34) | .708 |
| Father's SA | 0.98 (0.86, 1.13) | .815 | 0.93 (0.51, 1.69) | .813 |
| **External-cause mortality** | | |  |  |
| Mother's SA | 3.28 (2.14, 5.03) | <.001 | 2.18 (1.32, 3.62) | .003 |
| Father's SA | 1.94 (1.40, 2.68) | <.001 | 1.51 (1.05, 2.19) | .027 |
| Accidents |  |  |  |  |
| Mother's SA | 3.12 (1.88, 5.18) | <.001 | 2.24 (1.25, 4.01) | .007 |
| Father's SA | 2.02 (1.39, 2.93) | <.001 | 1.59 (1.04, 2.41) | .031 |
| Homicides |  |  |  |  |
| Mother's SA | 1.84 (0.43, 7.82) | .411 | 0.90 (0.19, 4.28) | .893 |
| Father's SA | 2.15 (0.90, 5.12) | .083 | 2.07 (0.78, 5.48) | .141 |
| Model 1 controls for offspring's sex and birth year, mother's age at offspring's birth and quadratic mother's age at offspring's birth. | | | | |
| Model 2 controls for Model 1 and father's income, co-parent’s SA, and mother and father's education, immigrant status, and severe mental disorders. | | | | |

| Table S13. All-cause and cause-specific mortality in ages 1–9 by lifetime parental substance abuse (SA). | | | | |
| --- | --- | --- | --- | --- |
|  | Model 1 | | Model 2 | |
|  | HR (95% CI) | p | HR (95% CI) | p |
| **All-cause mortality** | |  |  |  |
| Mother's SA | 1.28 (1.12, 1.46) | <.001 | 1.14 (0.99, 1.32) | .071 |
| Father's SA | 1.20 (1.12, 1.30) | <.001 | 1.04 (0.96, 1.14) | .308 |
| **Natural-cause mortality** | | |  |  |
| Mother's SA | 1.09 (0.92, 1.28) | .342 | 1.07 (0.89, 1.28) | .492 |
| Father's SA | 1.03 (0.93, 1.13) | .567 | 0.93 (0.83, 1.03) | .156 |
| Infectious diseases | |  |  |  |
| Mother's SA | 1.66 (1.16, 2.40) | .006 | 1.68 (1.14, 2.48) | .009 |
| Father's SA | 1.37 (1.09, 1.72) | .008 | 1.14 (0.88, 1.48) | .332 |
| Neoplasms | |  |  |  |
| Mother's SA | 0.83 (0.58, 1.17) | .282 | 0.78 (0.53, 1.14) | .201 |
| Father's SA | 0.95 (0.79, 1.14) | .590 | 0.89 (0.73, 1.08) | .248 |
| Congenital malformations | | |  |  |
| Mother's SA | 1.04 (0.73, 1.47) | .835 | 1.21 (0.44, 3.34) | .708 |
| Father's SA | 1.03 (0.85, 1.25) | .765 | 0.93 (0.51, 1.69) | .813 |
| **External-cause mortality** | | |  |  |
| Mother's SA | 1.85 (1.50, 2.28) | <.001 | 1.34 (1.05, 1.69) | .017 |
| Father's SA | 1.75 (1.53, 1.98) | <.001 | 1.38 (1.19, 1.59) | <.001 |
| Accidents |  |  |  |  |
| Mother's SA | 1.72 (1.37, 2.16) | <.001 | 1.24 (0.96, 1.61) | .098 |
| Father's SA | 1.78 (1.56, 2.04) | <.001 | 1.45 (1.24, 1.68) | <.001 |
| Homicides |  |  |  |  |
| Mother's SA | 3.67 (2.05, 6.58) | <.001 | 2.68 (1.31, 5.48) | .007 |
| Father's SA | 1.17 (0.69, 2.01) | .555 | 0.72 (0.38, 1.36) | .315 |
| Model 1 controls for offspring's sex and birth year, mother's age at offspring's birth and quadratic mother's age at offspring's birth. | | | | |
| Model 2 controls for Model 1 and father's income, co-parent’s SA, and mother and father's education, immigrant status, and severe mental disorders. | | | | |
